# An illustrated key to the genera and subgenera of the Recent azooxanthellate Scleractinia (Cnidaria, Anthozoa), with an attached glossary

**DOI:** 10.3897/zookeys.227.3612

**Published:** 2012-10-05

**Authors:** Stephen D. Cairns, Marcelo V. Kitahara

**Affiliations:** 1 Department of Invertebrate Zoology, National Museum of Natural History, Smithsonian Institution, Washington, DC 20560, USA; 2 Centro de Biologia Marinha, Universidade de São Paulo, São Sebastião, SP 11600-000, Brazil

**Keywords:** Azooxanthellate, Illustrated Key, Genera, Glossary, Scleractinia

## Abstract

The 120 presently recognized genera and seven subgenera of the azooxanthellate Scleractinia are keyed using gross morphological characters of the corallum. All genera are illustrated with calicular and side views of coralla. All termes used in the key are defined in an illustrated glossary. A table of all species-level keys, both comprehensive and faunistic, is provided covering the last 40 years.

## Introduction

The ready identification of azooxanthellate Scleractinia (determined herein by depth of occurrence and previously published observations) to the genus and species levels has been hampered by a lack of a comprehensive key to the genera as well as a lack of species level keys. For instance, the last comprehensive set of keys to the genera was published by Vaughan and Wells (1943) almost 70 years ago, and relied in part on microstructural characters that were both hard to observe (requiring thin sectioning) and interpret. Since then the number of Recent azooxanthellate genera and species has almost doubled, and new observations on apozooxanthellate species (species that have facultative symbiosis with zooxanthellae) are also available. Furthermore, what keys exist to the species level of various taxa or geographic regions are scattered throughout the literature and of variable quality (Table 1). In this Table, tabular keys are included, as they provide as much if not more information than a conventional dichotomous key. As result of the application of molecular data (e.g. Fukami et al. 2008, Kitahara et al. 2010a, Huang et al. 2011, Stolarski et al. 2011, Arrigoni et al. 2012), the higher taxonomic ranks of the order Scleractinia were shown to be polyphyletic. As such, a key to this taxonomic rank seems premature. Thus, it is the purpose of this paper to provide a single, comprehensive, illustrated key to the presently recognized 120 azooxanthellate scleractinian genera and 7 additional subgenera. We constructed the key using gross morphological characteristics of the corallum, which, when used in conjunction with the glossary and illustrations, we hope will provide a guide to the proper genus identification. But one must keep in mind that this key, as most, will not necessarily supply a definitive identification of the genus, as its use depends on the interpretation of the characters as well as the variation of that character state. We have used many of the dichotomies published by Vaughan and Wells (1943), but avoided the microstructural characters, and updated the taxa. Whereas microstructure is undoubtedly a valuable set of characters to define genera, in most cases it is not necessary to identify the genera. Among the 120 extant azooxanthellate scleractinian genera, 74 are illustrated with its type species (~61%). Within the remaining 46 genera, 20 (~16,6%) have an extinct species as type, represented by a fossil coral. For them and the remaining 26 genera, the illustrated species present very well the most important morphological characters of their respective genus.

**Table 1.** Previously published keys to azooxanthellate taxa, divided as comprehensive keys to all taxa with in a monophyletic taxon, and partial (faunistic) keys of species. Taxa listed alphabetically by taxon name. Tabular keys (T) are included.

**Comprehensive keys**	
*Anthemiphyllia*, species (T)	Cairns (1999: 290)
*Asterosmilia*, species (T)	Cairns and Wells (1987: 38)
*Aulocyathus*, species	Cairns (1999: 104)
*Caryophyllia*, species (T)	Cairns (1991: 12)
*Caryophyllia*, species	Kitahara et al. (2010b: 112)
*Conocyathus*, species	Cairns (2004a: 290)
*Crispatotrochus*, species	Kitahara and Cairns (2008: 62)
*Deltocyathus*, species	Kitahara and Cairns (2009: 236)
Dendrophylliidae, genera (T)	Cairns (2001: 5)
Flabellidae, genera (T)	Zibrowius (1974: 26); Cairns (1989: 45)
Guyniidae, genera (T)	Cairns (1989: 41); Stolarski (2000: 23)
*Javania*, species	Cairns (2004b: 10)
Micrabaciidae, genera	Cairns (1989: 13)
*Placotrochides*, species	Cairns (2004a: 307)
Scleractinia, families and genera	Vaughan and Wells (1943)
*Stephanophyllia*, species	Cairns (1989: 21)
*Trochocyathus (Aplocyathus*), species (T)	Cairns (1999: 85)
Turbinoliidae, genera	Cairns (1988a: 711; 1989: 25; 1997: 5 [T]); Filkorn (1994: 44)
**Faunistic keys**	
*Astrangia*, E. Pacific	Durham and Barnard (1952: 60)
Azooxanthellate Scleractinia, Antarctica	Cairns (1990: 18 [book])
Azooxanthellate Scleractinia, E. Gulf of Mexico	Cairns (1977a: 5)
Azooxanthellate Scleractinia, New Zealand	Squires and Keyes (1967: 13); Tracey et al. (2012)
Azooxanthellate Scleractinia, NE Pacific	Cairns (1994: 13)
Azooxanthellate Scleractinia, NW Pacific	Cairns (1994: 75)
Azooxanthellate Scleractinia, S. Australia	Cairns and Parker (1992: 4)
Azooxanthellate Scleractinia, Cold Temp. NE Atl.	Cairns (1981: 3)
Azooxanthellate Scleractinia, Brazil	Kitahara (2007: 510)
*Balanophyllia*, W. Atlantic	Cairns (1977b: 133)
*Balanophyllia*, Japan	Ogawa et al. (1998: 145 [in Japanese])
*Balanophyllia*, W. Atlantic (T)	Cairns (2000: 163)
*Caryophyllia*, New Zealand	Cairns (1995: 43)
*Caryophyllia*, W. Atlantic	Cairns (1979: 46)
*Caryophyllia*, W. Pacific	Cairns and Zibrowius (1997: 87, 96)
*Caryophyllia* and *Premocyathus*, Japan	Ogawa et al. (1999: 115 [in Japanese])
*Conotrochus* and *Trochocyathus*, Japan	Ogawa et al. (2003: 57 [in Japanese])
*Culicia*, Australia	Cairns (2004a: 274)
*Deltocyathus*, W. Atlantic	Cairns (1979: 91)
*Deltocyathus*, W. Pacific	Cairns and Zibrowius (1997: 121)
*Dendrophyllia*, Japan	Ogawa and Takahashi (1995: 25 [in Japanese])
*Flabellum*, New Zealand	Cairns (1995: 96)
*Flabellum*, Japan	Ogawa and Takahashi (2005: 56 [in Japanese])
*Fungiacyathus*, W. Pacific (T)	Cairns (1989: 6, 7; 1999: 55)
*Fungiacyathus*, Japan	Ogawa and Takahashi (2004: 11 [in Japanese])
*Heterocyathus*, W. Pacific	Hoeksema and Best (1991: 222)
*Heterocyathus*, Japan	Ogawa and Takahashi (2008: 248 [in Japanese])
*Heteropsammia*, W. Pacific	Hoeksema and Best (1991: 222)
*Heteropsammia*, Japan	Ogawa and Takahashi (2008: 248 [in Japanese])
*Madracis*, W. Atlantic	Wells (1973: 19)
*Paracyathus* and *Polycyathus*, Japan	Ogawa et al. (2000: 55 [in Japanese])
*Trochocyathus*, W. Pacific	Cairns and Zibrowius (1997: 105)
*Truncatoflabellum*, W, Pacific	Cairns (1989a: 62)
*Truncatoflabellum*, SW Indian Ocean	Cairns and Keller (1993: 264)
*Truncatoflabellum*, Australia (T)	Cairns (1998: 397)
*Truncatoflabellum*, Japan	Ogawa (2006: 13 [in Japanese])
*Tubastraea*, Red Sea	Scheer and Pillai (1983: 173)
*Tubastraea*, Galapagos	Cairns (1991: 27)
*Tubastraea*, Japan	Ogawa and Takahashi (1993: 97 [in Japanese])
*Turbinoliidae*, Japan	Ogawa et al. (2002: 27 [in Japanese])

## Methods

Some genera are keyed two or even three times because of the variation within those genera regarding the characters used in the key. In theory, all variations of that genus will be correctly keyed. Although most couplets are dichotomous, some are polychotomous, such as the columella or colony shape, which allows the reader to clearly see the multiple states of a particular character.

Although it would be desirable to follow the generic key with keys to all of the approximately 720 azooxanthellate species, it is a simple fact that not many species level keys have been published. Those that have been published in the last 35 years are listed in Table 1, separated as to whether they are keys to all of the taxa within a monophyletic taxon (comprehensive) or to a more limited fauna of a region (faunistic). Keys made before 1970 were found to be, in general, not up to date and are thus not included. It should be noted that fully one-third of the genera (40) are monotypic, and thus do not require a key following a correct genus identification, and another 22 genera have but two species. Finally, although they do not include keys, the treatises of Wells (1956) and Chevalier and Beauvais (1987) include diagnoses of all genera, including those represented only by extinct species, and thus provide a rich source of taxonomic information.

Other sources of useful taxonomic information include a list of all extant Recent scleractinian species as of 1999 (Cairns et al. 1999), which also includes a rough indication of their geographic range. The azooxanthellate component of this list is kept up to date as an on-line resource (www.lophelia.org/online-appendices), which now includes junior synonyms and depth ranges of the species, and authors of the genera. A list of the 120 azooxanthellate genera, their authorship, and bathymetric ranges was also published in Roberts et al. (2009: Table 2.7)

Geographic ranges within brackets in the key are not meant to be considered as distinguishing characters, but simply informational, which may nonetheless hint at an incorrect identification. Abbreviations: Ant. = Antarctic or Subantarctic, Atl. = Atlantic, IP = Indo Pacific, IWP = Indo-West Pacific, Pac. = Pacific, SubAnt = Subantarctic; Cosmopolitan implies occurrence in all three oceans as well as Subantarctic and/or Antarctic. Museums and Institutions acronyms: AM = Australian Museum (Sydney); AU = Auckland University Museum (Auckland); CSIRO = Commonwealth Scientific and Industrial Research Organisation (Hobart); JCU = James Cook University (Townsville); MNHN = Muséum national d’Histoire naturelle (Paris); SBMNH = Santa Barbara Natural History Museum (Santa Barbara); SIO = Scripps Institute of Oceanography (San Diego); NZOI = New Zealand Oceanographic Institution (now the National Institute of Water and Atmospheric Research) (Wellington); USNM = United States National Museum (now the National Museum of Natural History, Smithsonian) (Washington, D.C.); YPM = Yale Peabody Museum (New Heaven).

Useful sources for more information about definitions of terms used in the glossary include: Wells (1956), and Cairns (1981, 1989, 1994).

### Key to the Genera and Subgenera of the Recent Azooxanthellate Scleractinia

(An asterisk indicates genera that have azooxanthellate and zooxanthellate representatives)

**Table:** 

1a	Corallum colonial	2
1b	Corallum solitary	43
2a	Corallum free of attachment (recumbent, usually curved with a broken or open base, or globular)	3
2b	Corallum firmly attached (arborescent, bushy, encrusting, or reptoid)	5
3a	Corallum recumbent (composed of a large primary corallite from which smaller buds originate); no sipunculid commensalism	4
3b	Corallum globular; pores in lateral base of colony associated with commensal sipunculid	[IWP] Heteropsammia* (in part) Plate 1, Figures A–B
4a	Corallum not porous (solid); septa arranged normally	[Atl. + IP] *Anomocora* Plate 1, Figures C–D
4b	Corallum, especially septa porous; septa arranged in a Pourtalès Plan	[Atl. + IWP] *Eguchipsammia* Plate 1, Figures E–F
5a	Corallum arborescent or bushy	6
5b	Corallum encrusting or reptoid	27
6a	Branching intratentacular	7
6b	Branching extratentacular	9
7a	Equal distomadeal budding	8
7b	Unequal monostomaeous budding	[Cosmopolitan] *Lophelia* Plate 1, Figures G–H
8a	Texture of corallum rough (like sandpaper), resulting from a porous theca; septa arranged in a weak Pourtalès Plan	[W. Pac.] *Dichopsammia* Plate 1, Figures I–J
8b	Texture of corallum smooth or costate, solid; septa arranged normally	Cosmopolitan] *Solenosmilia* Plate 1, Figures K–L
9a	Septal symmetry decameral or octameral, septa in only one cycle; columella styliform	[Atl. + IP] Madracis* (in part) Plate 2, Figures A–B
9b	Septal symmetry hexameral, septa arranged in multiple cycles; columella papillose, fascicular or absent	10
10a	Texture of theca and septa rough (like sandpaper), resulting from a porous theca	11
10b	Texture of theca smooth, granular, or ridged (solid)	14
11a	Septa arranged in a Pourtalès plan	12
11b	Septa arranged normally	13
12a	Corallum small (bushy), most corallites budding from a common basal coenosteum or from the edge zone of corallites that originate from the basal coenosteum	[Atl. + Pac.] *Cladopsammia* Plate 2, Figures C–D
12b	Corallum large (bushy to arborescent), with multiple successive generations of budding forming an erect colony	[Atl. + IP] *Dendrophyllia* Plate 2, Figures E–F
13a	Corallum porosity only apparent near calicular edge; found in deep-water: 110-2165 m	[Atl. + IWP] *Enallopsammia* Plate 2, Figures G–H
13b	Corallum porosity uniform: shallow-water: 0-110 m	[Atl. + IP] *Tubastraea* (in part) Plate 2, Figures I–J
14a	Columella absent	15
14b	Columella present (papillose, trabecular or fascicular)	16
15a	Corallum large (arborescent), with numerous budding cycles, adjacent corallites often linked with hollow, tubular coenosteal bridges; tabular endothecal dissepiments common	[I–P + Subant.] *Goniocorella* Plate 2, Figures K–L
15b	Corallum a small bush, corallites originating from a common basal coenosteum or from the sides of other corallites and from relatively few budding cycles; endothecal dissepiments not prominent	[E. Atl. + New Zealand] *Hoplangia* (in part) Plate 5, Figures L–M
16a	Columella fascicular	17
16b	Columella papillose or trabecular	18
17a	Pali before septa of third cycle (P3)	[N. Atl.] *Pourtalosmilia* Plate 3, Figures A–B
17b	Pali absent	[W. Pac.] *Confluphyllia* Plate 3, Figures C–D
18a	Columella trabecular, composed of slender ( flattened laths); corallum never with more than 4 generations of budding	[Atl. + W. Pac.] *Coenosmilia* Plate 3, Figures E–F
18b	Columella papillose (composed of rods); corallum composed of many generations of budding	19
19a	Axial septal edges dentate	[W. Pac.] *Sympodangia* Plate 3, Figures G–H
19b	Axial septal edges smooth	20
20a	Pali absent	[Cosmopolitan] *Madrepora* (in part) Plate 3, Figures I–J
20b	Pali present	21
21a	Pali arranged in multiple crowns before septa of all but last cycle; axial edge of septa minutely dentate	22
21b	Pali arranged in two crowns before S2 and S3 or S1-3; axial edges of septa smooth	24
22a	Coenosteum costate	[Atl. + Pac.] *Cladocora* Plate 3, Figures K–L
22b	Coenosteum not costate	23
23a	Axial corallite associated with each branch	[SW Pac.] *Petrophyllia* Plate 4, Figures A–B
23b	Axial corallites absent	[Atl. + Pac.] Oculina* Plate 4, Figures C–D
24a	P1-3 arranged in two palar crowns	[IWP] *Cyathelia* Plate 4, Figures E–F
24b	One palar crown of P2 or P3	25
25a	Only P2 present	26
25b	Only P3 present	[SW Atl. + E. Pac.] *Bathelia* Plate 4, Figures G–H
26a	Columella massive	[SE Atl.] *Sclerhelia* Plate 4, Figures I–J
26b	Columella rudimentary	[Cosmopolitan] *Madrepora* (in part) Plate 4, Figures K–L
27a	Septal symmetry decameral or octameral, septa in only one cycle; columella styliform	[Atl. + IP] Madracis* (in part) Plate 5, Figures A–B
27b	Septal symmetry hexameral, septa arranged in multiple cycles; columella papillose, fascicular, spongy, lamellar or absent	28
28a	Texture of corallum rough (like sandpaper), resulting from a porous theca	29
28b	Texture of corallum smooth or costate, solid	31
29a	Corallum increases by stoloniferous budding (reptoid), the connection among corallites often obscured, thus sometimes appearing to be solitary; Pourtalès Plan present	[W. Atl. + IP] *Rhizopsammia* Plate 5, Figures C–D
29b	Corallum increases by budding from a common basal coenosteum, the connection among polyps quite evident; septa normally inserted	30
30a	Columella massive; epitheca surrounds each corallite	[E. Atl.] *Astroides* Plate 5, Figures E–F
30b	Columella of moderate to small size; epitheca lacking	[Atl. + IP] *Tubastraea* (in part) Plate 5, Figures G–H
31a	Columella absent	32
31b	Columella present	33
32a	Corallites united by thin basal stolons (reptoid)	[Atl. + IWP] *Thalamophyllia* Plate 5, Figures I–K
32b	Corallites bud from a common basal coenosteum	[E. Atl. + New Zealand] *Hoplangia* (in part) Plate 5, Figures L–M
33a	Axial edges of some or all cycles of septa finely dentate or beaded	(Rhizangiidae) 34
33b	Axial edges of all septa smooth	38
34a	Thin epitheca encircles corallites; axial edges of S1-2 smooth, sometimes lobate (but inner edges of S3-4 dentate)	35
34b	Epitheca absent; axial edges of all septa dentate	36
35a	Corallite base polycyclic; one crown of large P3	[Atl.+ Pac.] *Colangia* Plate 6, Figures A–B
35b	Corallite base monocyclic; pali, if present, of uniform size	[IP] *Culicia* Plate 6, Figures C–D
36a	Corallite base polycyclic; pali absent	[IP] *Oulangia* Plate 6, Figures E–F
36b	Corallite base monocyclic; pali before septa of all but last cycle	37
37a	Corallum stoloniferous (reptoid) or cerioid; peritheca absent	[Atl. + IP] Astrangia* Plate 6, Figures G–H
37b	Corallum massive (subramose); peritheca unite corallites	[Indian] *Cladangia* Plate 6, Figures I–J
38a	Pali or paliform lobes on axial edges of septal of all but last cycle	39
38b	Pali or paliform lobes present only on septa of penultimate cycle (usually P3)	41
39a	Corallum stoloniferous (reptoid)	[IWP] *Rhizosmilia* Plate 6, Figures K–L
39b	Corallites bud from a common basal coenosteum	40
40a	Corallites monocyclic; pali before septa of all but last cycle, and all of approximately the same size	[IWP] *Polycyathus* Plate 7, Figures A–B
40b	Corallites polycyclic; pali before septa of all but last cycle, those of P3 crown much larger than others	[W. Atl.] *Phacelocyathus* Plate 7, Figures C–D
41a	Columella fascicular	42
41b	Columella trabecular	[Atl. + IP] *Phyllangia* Plate 7, Figures E–F
42a	Occurrence of pali variable: usually P4, occasionally also P3, occasionally absent	[E. Pac.] *Bathycyathus* Plate 7, Figures G–H
42b	Pali in one crown before septa of third cycle (P3)	[Atl. + Pac.] *Coenocyathus* Plate 7, Figures I–J
43a	Corallum firmly attached (fixed)	44
43b	Corallum unattached (free)	67
44a	Theca granular, the granules usually occurring on longitudinally oriented costae	45
44b	Theca smooth (epithecate or stereome-reinforced), sometimes with fine transverse ridges encircling the theca	53
44c	Theca and septa porous, although in some genera a smooth epitheca may cover the basal portion of the corallum	61
44d	Theca absent (corallum discoidal)	[E. Pac.] *Nomlandia* Plate 7, Figure K
45a	Columella papillose	46
45b	Columella fascicular	51
45c	Columella absent	52
45d	Columella labyrinthiform	[Atl. + IP] *Labyrinthocyathus* Plate 8, Figures A–B
46a	Pali or paliform lobes absent; base polycyclic	[W. Atl. + W. Pac.] *Oxysmilia* Plate 8, Figures C–D
46b	Pali or paliform lobes present; base monocyclic	47
47a	Coralla usually arranged in pseudocolonial assemblages	[W. Pac.] *Lochmaeotrochus* Plate 8, Figures G–H
47b	Coralla discrete	48
48a	Pali before S1-2 (P1, P2), indistinguishable from columellar elements	[W. Atl. + IWP] *Monohedotrochus* Plate 8, Figures E–F
48b	Pali before septa of all but last cycle; palar crowns discrete	49
49a	Multiple slender paliform lobes on axial edge of every lower cycle septum, not arranged in crowns	[Atl. + IP] *Paracyathus* Plate 8, Figures I–J
49b	Two crowns of discrete pali or paliform lobes (P1+P2 and P3), only one palus or paliform lobe per septum	50
50a	True pali present, the P1-2 smaller than P3 but not significantly	[Atl. + IP] *Trochocyathus (Trochocyathus)* (in part) Plate 8, Figures K–L
50b	Paliform lobes present, the P1-2 much smaller than the broad P3	[W. Atl. + W. Pac.] *Vaughanella* Plate 9, Figures A–B
51a	Pali before septa of penultimate cycle	[Cosmopolitan] *Caryophyllia* (*Caryophyllia*) (in part) Plate 9, Figures C–D
51b	Pali absent	[Cosmopolitan] *Crispatotrochus* Plate 9, Figures E–F
52a	Corallum base monocentric; epitheca lacking; calice elliptical in outline; menianes lacking	[Cosmopolitan] *Desmophyllum* Plate 9, Figures G–H
52b	Corallum polycentric; transverse epithecal bands near corallum base; calicular outline modified by calicular extensions; menianes on septal faces	[W. Pac.] *Dactylotrochus* Plate 9, Figures I–J
53a	Columella absent or simply a rudimentary fusion of lower axial edges of major septa deep in fossa	54
53b	Columella present (papillose, fascicular or labyrinthiform)	57
54a	Pedicel reinforced (thickened) with stereome deposits	[Cosmopolitan] *Javania* Plate 9, Figures K–L
54b	Pedicel reinforced with hollow rootlets, most easily seen in cross section of base or pedicel, or in a damaged corallum	55
55a	Rootlets non-contiguous with pedicel, 2-20 adventitious rootlets anchoring the corallum	[IWP] *Rhizotrochus* Plate 10, Figures A–B
55b	Rootlets (symmetrical or asymmetrical in placement) contiguous with pedicel, forming an integral part of the lower corallum	56
56a	Calicular edge jagged	[W. Atl. + IP] *Polymyces* Plate 10, Figures C–D
56b	Calicular edge smooth	[E. Atl. + W. Pac.] *Monomyces* Plate 10, Figures E–F
57a	Columella papillose	58
57b	Columella fascicular	60
57c	Columella labyrinthiform	[W. Pac.] *Stolarskicyathus* Plate 10, Figures G–I
58a	Corallum base polycyclic; no notch between upper outer edges of septa and theca	59
58b	Base monocyclic, but may have an accessory basal rootlet; septal notch present	[W. Atl. + IWP] *Gardineria* Plate 10, Figures J–K
59a	Pali before septa of penultimate cycle	[Atl. + E. Pac.] *Concentrotheca* Plate 10, Figures L–M
59b	Paliform lobes present before septa of S1-2 (P1-2)	[E. Atl. + E. Pac.] *Ceratotrochus* Plate 11, Figures A–B
59c	Pali before septa of all but last cycle in two crowns	[Atl. + Pac.] *Tethocyathus* Plate 11, Figures C–D
60a	Corallum cylindrical and very small (calicular diameter less than 2 mm); a row of thecal spots or pores present in every interseptal region; octameral septal symmetry; only 1 columellar element	[Atl. + IWP] *Guynia* Plate 11, Figures E–G
60b	Corallum trochoid and larger (adult calicular diameter over 10 mm); thecal spots and pores lacking; hexameral symmetry; numerous columellar elements	[IWP] *Conotrochus* (in part) Plate 11, Figures H–I
61a	Septa arranged in a Pourtalès Plan	62
61b	Septa arranged normally	63
62a	Corallum base polycyclic; theca costate	[Cosmopolitan] Balanophyllia (Balanophyllia)* Plate 11, Figures J–K
62b	Corallum base monocyclic; theca hispid (not costate)	[W. Atl. + SW Pac.] *Thecopsammia* Plate 11, Figures L–M
63a	Columella absent or rudimentary	64
63b	Columella spongy	65
64a	Corallum trochoid; theca costate	[W. Atl.] *Trochopsammia* Plate 12, Figures A–B
64b	Corallum subcylindrical (sometimes scolecoid); theca uniformly hispid (not costate)	[S. Africa] *Pourtalopsammia* Plate 12, Figures C–D
65a	Costae absent; axial edges of all septa smooth; no endothecal dissepiments	[W. Atl.] *Bathypsammia* Plate 12, Figures E–F
65b	Costae granular or hispid; axial edges of higher cycle septa dentate to laciniate; endothecal dissepiments present in an elongate corallum	66
66a	Columella not discrete (merging with lower axial edges of septa); costae weakly granular …	[IP] *Endopsammia* Plate 12, Figures G–H
66b	Columella discrete; costae hispid	[E. Atl. + IWP] *Leptopsammia* Plate 12, Figures I–J
67a	Corallum unattached (free) in every growth stage (lacking transverse division)	68
67b	Corallum undergoes transverse division, resulting in a free anthocyathus stage with a basal scar, but with a fixed anthocaulus stage	102
68a	Corallum conical (ceratoid, trochoid or turbinate)	69
68b	Corallum bowl-shaped	87
68c	Corallum cupolate (theca horizontal with no surrounding vertical theca)	91
68d	Corallum cuneiform	(Turbinoliidae, in part) 97
68e	Corallum globular (pores in base of corallum associated with commensal sipunculid)	101
68f	Corallum cylindrical	[Atl. + IWP + Ant.] *Stenocyathus* Plate 12, Figures K–L
69a	Columella papillose	70
69b	Columella rudimentary or absent	78
69c	Columella fascicular	83
69d	Columella trabecular	86
69e	Columella styliform	[SW Pac.] *Turbinolia* Plate 13, Figures A–B
69f	Columella spongy	[W. Atl. + IWP] *Balanophyllia (Eupsammia)* Plate 13, Figures C–D
70a	Pali or paliform lobes present	71
70b	Pali and paliform lobes absent	[IWP] *Foveolocyathus* Plate 13, Figures E–F
71a	Pali before septa of second cycle (P2)	72
71b	Pali or paliform lobes before septa of all but last cycle	77
71c	Pali or paliform lobes before septa of third cycle (P3)	E. Atl. + IWP + Ant.] *Paraconotrochus* Plate 13, Figures G–H
72a	Theca bears numerous linear rows of spots, pits or thecal perforations	73
72b	Theca solid, not bearing spots, pits or perforations	75
73a	Theca perforate	[W. Atl. + W. Pac.] *Trematotrochus* Plate 13, Figures I–J
73b	Theca bears linearly arranged spots or pits	74
74a	A row of pits occurs in each interseptal space on inner theca; costae granular	[W. Pac.] *Endocyathopora* Plate 13, Figures K–L
74b	A row of white spots occurs in each interseptal space on outer theca; theca smooth (epithecate) or covered with hispid spines	[W. Atl.] *Pourtalocyathus* Plate 14, Figures A–B
75a	Theca bears serrate costae	76
75b	Theca smooth (epithecate)	[SW Pac.] *Lissotrochus* Plate 14, Figures C–D
76a	Theca covered with twice as many costae as septa	[SW Pac.] *Pleotrochus* Plate 14, Figures E–F
76b	Costae and septa of equal number	[W. Atl. + W. Pac.] *Cryptotrochus* Plate 14, Figures G–H
77a	Pali discrete, pairs of P3 fused into chevrons within each system; no parricidal budding	[W. Pac.] *Notocyathus* Plate 14, Figures I–J
77b	Multiple paliform lobes on all septa; parricidal budding common	[IWP] *Thrypticotrochus* Plate 14, Figures K–L
78a	Theca smooth (epithecate), costae not present	79
78b	Theca granular, costae present (twice the number of septa)	82
79a	Rows of thecal spots visible on theca	80
79b	Thecal spots lacking	81
80a	Twelve contiguous rootlets present in pedicel; parricidal budding absent	[W. Pac.] *Pedicellocyathus* Plate 15, Figures A–C
80b	Rootlets lacking; parricidal budding from parent fragment common	[Atl.] *Schizocyathus* Plate 15, Figures D–E
81a	Calicular edge smooth	[Cosmopolitan] *Flabellum (Flabellum)* Plate 15, Figures F–G
81b	Calicular edge jagged	[Cosmopolitan] *Flabellum (Ulocyathus)* Plate 15, Figures H–I
82a	Theca perforate; septa hexamerally arranged in 3 or 4 cycles	[IWP] *Conocyathus* Plate 15, Figures J–K
82b	Theca imperforate; only10 septa (6+4)	[SW. Pac.] *Holcotrochus* Plate 15, Figures L–M
83a	Pali before septa of penultimate cycle (usually P3)	84
83b	Pali absent	[E. Pac.] *Pseudocyathoceras* Plate 16, Figures A–B
84a	Thecal edge spines or crests present	85
84b	Thecal edge spines and crests absent	[Cosmopolitan] *Caryophyllia (Caryophyllia)*(in part) Plate 16, Figures C–D
85a	Base of corallum usually open, as though broken from parent through asexual budding	[Atl. + IWP] *Premocyathus* Plate 16, Figures E–F
85b	Base of corallum intact	[W. Pac.] *Caryophyllia (Acanthocyathus)* Plate 16, Figures G–H
86a	Theca costate; septal notch absent	[Atl. + IWP] *Dasmosmilia* Plate 16, Figures I–J
86b	Theca smooth; septal notch present	[E. Atl. + IWP + Ant.] *Aulocyathus* Plate 16, Figures K–M
87a	Paliform lobes on septa of all cycles; septal edges smooth	88
87b	Pali before septa of all but last cycle; septal edges smooth	90
87c	Pali before septa of third cycle (P3); septal edges smooth	[W. Pac.] *Ericiocyathus* Plate 17, Figures A–B
87d	Pali and paliform lobes absent; septal edges coarsely dentate	[W. Atl. + IWP] *Anthemiphyllia* (in part) Plate 17, Figures C–D
88a	Lower outer edge of corallum bears tubercles or spines on the C1 or C1-2	89
88b	Tubercles and spines absent	[Atl. + IWP] *Stephanocyathus (Stephanocyathus)* Plate 17, Figures E–F
89a	Six long C1 spines on lower outer edge of corallum	[IWP] *Stephanocyathus (Acinocyathus)* Plate 17, Figures G–H
89b	Twelve to 18 short spines or tubercles on lower outer edge of corallum	[W. Atl. + IWP] *Stephanocyathus (Odontocyathus*) Plate 17, Figures I–J
90a	Six C1 spines on lower outer edge of corallum	[W. Pac.] *Trochocyathus (Aplocyathus)* Plate 17, Figures K–M
90b	Costal spines absent	[Atl. +Pac.] *Deltocyathoides* Plate 18, Figures A–C
91a	Costae alternate in position with septa; higher cycle septa increase by bifurcation; thecal base perforate	(Micrabaciidae) 92
91b	Costae continuous with septa; higher cycle septa increase by adding additional cycles; base imperforate	95
92a	Septa rudimentary, composed of a series of tall spines	[Cosmopolitan] *Leptopenus* Plate 18, Figures D–E
92b	Septa lamellar	93
93a	Marginal shelf present; columella spongy	94
93b	Marginal shelf absent; columella solid	[IWP] *Stephanophyllia* Plate 18, Figures F–H
94a	Septa highly porous	[IWP] *Letepsammia* Plate 18, Figures I–K
94b	Septa essentially imperforate, porous only at points at which septa bifurcate	[IWP] *Rhombopsammia* Plate 18, Figures L–N
95a	Synapticular platelets absent; corallum robust; upper septal edges smooth	[Atl. + IP] *Deltocyathus* Plate 18, Figures O–Q
95b	Synapticular platelets brace adjacent septa; corallum fragile; upper septal edges bear slender elongate spines	96
96a	Corallum with five cycles of septa (96 septa)	[Atl. + IWP. + Ant.] *Fungiacyathus (Fungiacyathus)* Plate 19, Figures A–C
96b	Corallum with four cycles of septa (48 septa)	[Cosmopolitan] *Fungiacyathus (Bathyactis)* Plate 19, Figures D–F
97a	Thecal edge crests present	98
97b	Thecal edge crests absent	100
98a	Pali absent	99
98b	Pali present, before septa of all but last cycle	[IWP] *Tropidocyathus* Plate 19, Figures G–H
99a	Twice as many costae as septa	[W. Pac.] *Alatotrochus* Plate 19, Figures I–J
99b	Equal number of costae and septa	[IWP] *Platytrochus* Plate 19, Figures K–M
100a	Columella lamellar; pali absent	[Atl. + Pac.] *Sphenotrochus* Plate 19, Figures N–O
100b	Columella papillose; pali before septa of all but last cycle	[IWP] *Cyathotrochus* Plate 20, Figures A–B
101a	Theca imperforate (although septa may be perforate)	[IWP + W. Atl.] Heterocyathus* Plate 20, Figures C–E
101b	Theca and septa perforate	[IWP] Heteropsammia* (in part) Plate 20, Figures F–H
102a	Columella papillose	103
102b	Columella spongy	108
102c	Columella a solid fusion in center of calice	109
102d	Columella absent	110
102e	Columella fascicular	111
102f	Columella lamellar	[IWP] *Placotrochus* Plate 20, Figures I–J
102g	Columella trabecular	[Atl. + IWP] *Placotrochides* Plate 20, Figures N–O
103a	Pali before septa of all but last cycle	104
103b	Pali before S1-2 (P1-2)	106
103c	Pali before S2 (P2)	[SW Pac.] *Kionotrochus* Plate 21, Figures A–B
103d	Pali absent	[W. Atl. + IWP] *Anthemiphyllia* (in part) Plate 21, Figures C–E
104a	Six long C1 spines on lower outer edge of corallum	[W. Pac.] *Bourneotrochus* Plate 21, Figures F–H
104b	Thecal spines absent, although corallum may bear thecal edge crests	105
105a	Corallum small (calicular diameter usually less than 5 mm); higher cycle septa bend toward and fuse with adjacent lower cycle septa	[Atl. + W. Pac.] *Peponocyathus* Plate 21, Figures I–K
105b	Corallum larger (calicular diameter usually over 10 mm); higher cycle septa independent	[W. Pac.] *Trochocyathus (Trochocyathus)* (in part) Plate 21, Figures L–N
106a	Septa alternate in position with costae; thecal spots absent	107
106b	Septa correspond to costae; lines of thecal spots present	[W. Pac.] *Temnotrochus* Plate 21, Figures O–P
107a	Corallum cuneiform in shape, sometimes with basal thecal spurs (fish-tail morphology)	[W. Pac.] *Idiotrochus* Plate 22, Figures A–C
107b	Corallum (anthocyathus) discoidal to bowl-shaped, without thecal spurs	.[W. Pac.] *Dunocyathus* Plate 22, Figures D–E
108a	Septa arranged in a Pourtalès Plan; thecal edges often crested	[IP] *Endopachys* Plate 22, Figures F–G
108b	Septa normally arranged; thecal edges rounded	[SW Pac.] *Notophyllia* Plate 22, Figures H–J
109a	Multiple paliform lobes on septa of all but last cycle; thecal spots absent; corallum discoidal to cupolate in shape	[Indian] *Australocyathus* Plate 22, Figures K–L
109b	Pali and paliform lobes absent; linear rows of thecal spots present; corallum compressed-cylindrical	[W. Pac.] *Truncatoguynia* Plate 22, Figures M–N
110a	Buds propagate from thecal edges of corallum	[W. Pac.] *Blastrotrochus* Plate 23, Figures A–B
110b	No budding from thecal edges	[E. Atl. +IWP + Ant.] *Truncatoflabellum* Plate 23, Figures C–F
111a	Pali before third cycle septa; theca costate	[Cosmopolitan] *Caryophyllia (Caryophyllia*) (in part) Plate 23, Figures G–H
111b	Paliform lobes before second cycle septa; epithecate	[SW Pac.] *Falcatoflabellum* Plate 20, Figures K-M

## Figures and Tables

**Figure 1.**
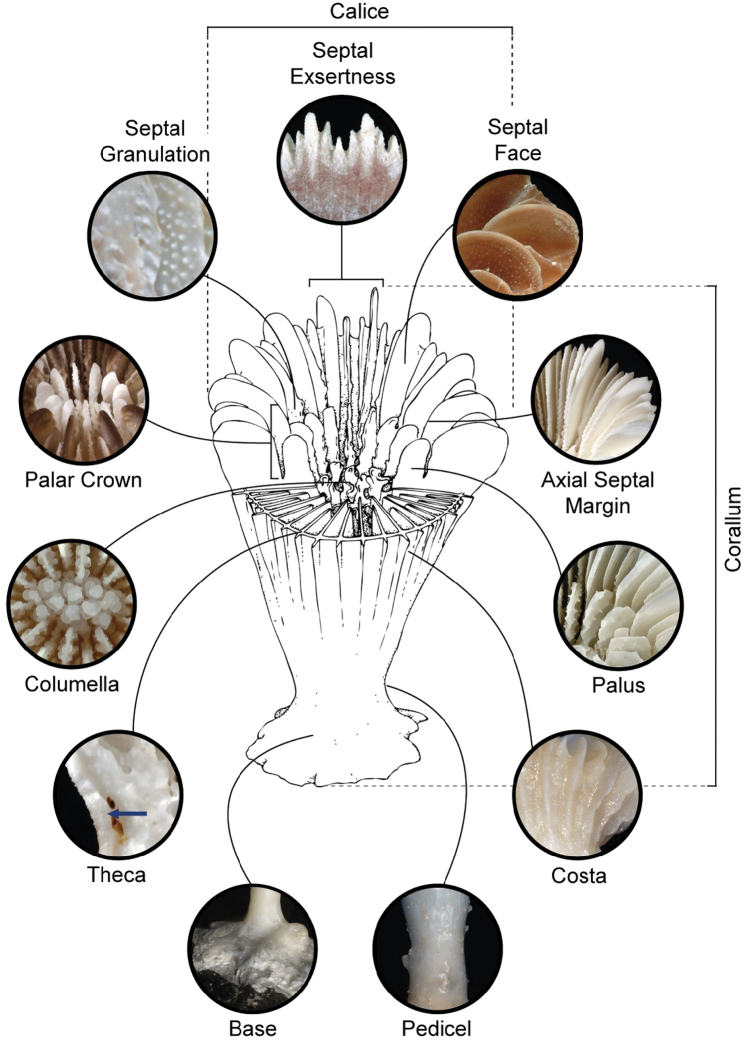
Cutaway diagram of a species of *Caryophyllia* illustrating the basic morphological features of an attached, solitary scleractinian (Modified from Cairns 1994). Small circular photos are from different scleractinian species and are intended to illustrate basic morphological characters used in the taxonomy of the group.

**Plate 1.**
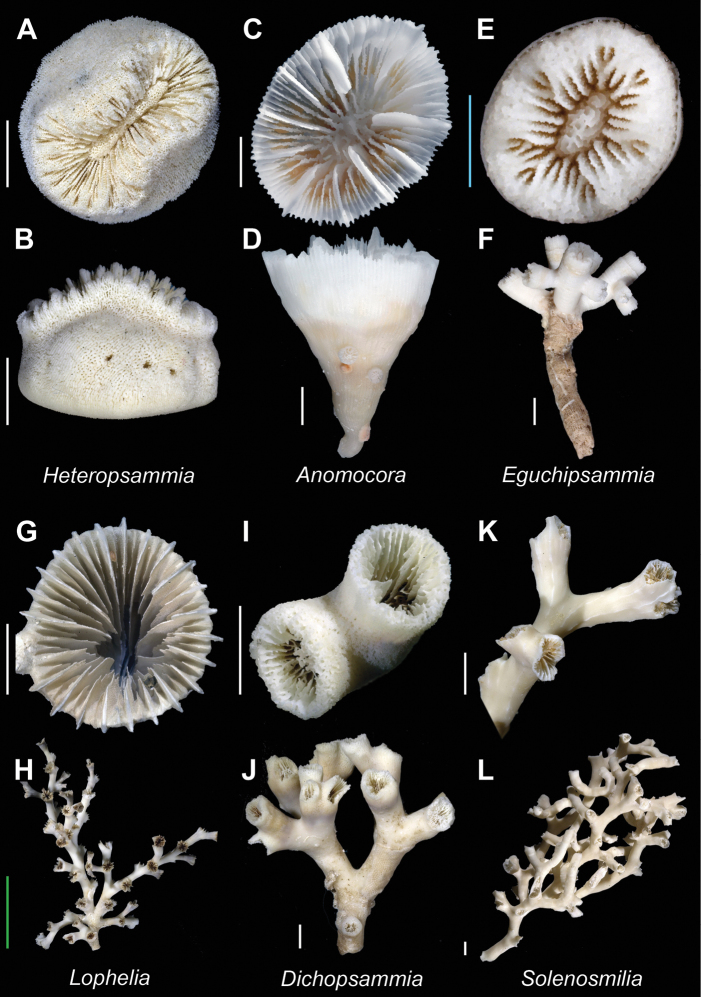
***Heteropsammia cochle*aA** (USNM 97652) and **B** (USNM 73772): Calicular and lateral view respectively; *Anomocora gigas* (MNHN uncatalogued, Terrasses stn. CP3091) **C** and **D** Calicular and lateral view respectively; *Eguchipsammia fistula* (USNM uncatalogued, Norfolk 2 stn. 2024) **E** and **F** Calicular and colony view respectively; ***Lophelia pertus*a**(USNM 1071877) **G** and **H** Calicular and colony view respectively; ***Dichopsammia granulos*a**(USNM 15847, holotype) **I** and **J** Calicular and colony view respectively; ***Solenosmilia variabili*s**(USNM 47426) **K** and **L** Distal branch and colony view respectively. Scale bars: blue = 1 mm; white = 5 mm; green = 50 mm. Bold face indicates type species for the genus.

**Plate 2.**
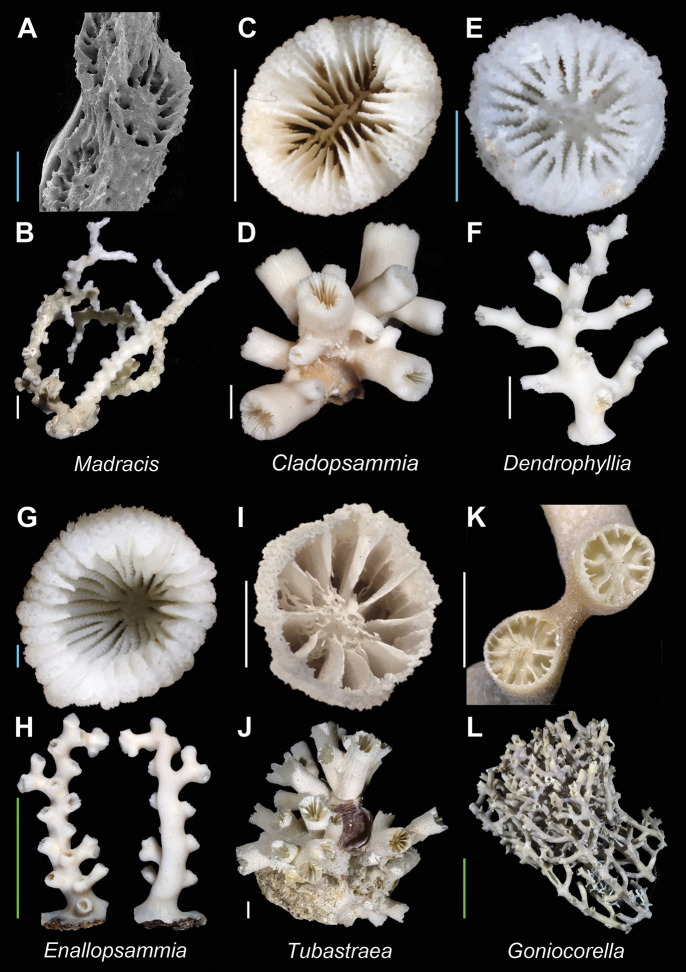
***Madracis asperula*A** (SEM, USNM 99068) and **B** (USNM 99056): Calicular and colony view respectively; *Cladopsammia* sp. (USNM uncatalogued, Norfolk 2 stn. 2023)**C** and **D** Calicular and colony view respectively; *Dendrophyllia alcocki* (USNM uncatalogued, Norfolk 2 stn. 2135)**E** and **F** Calicular and colony view respectively; *Enallopsammia rostrata* (USNM uncatalogued, Norfolk 2 stn. DW 2056) **G** and **H** Calicular and colony (calicular and acalicular side) view respectively; ***Tubastraea coccine*a**(USNM 46973) **I** Calicular view; *Tubastraea diaphana* (USNM 83677) **J** Colony view; ***Goniocorella dumos*a**(USNM 47505) **K** and **L** Calicular and colony view respectively. Scale bars: blue = 1 mm; white = 5 mm; green = 50 mm. Bold face indicates type species for the genus.

**Plate 3.**
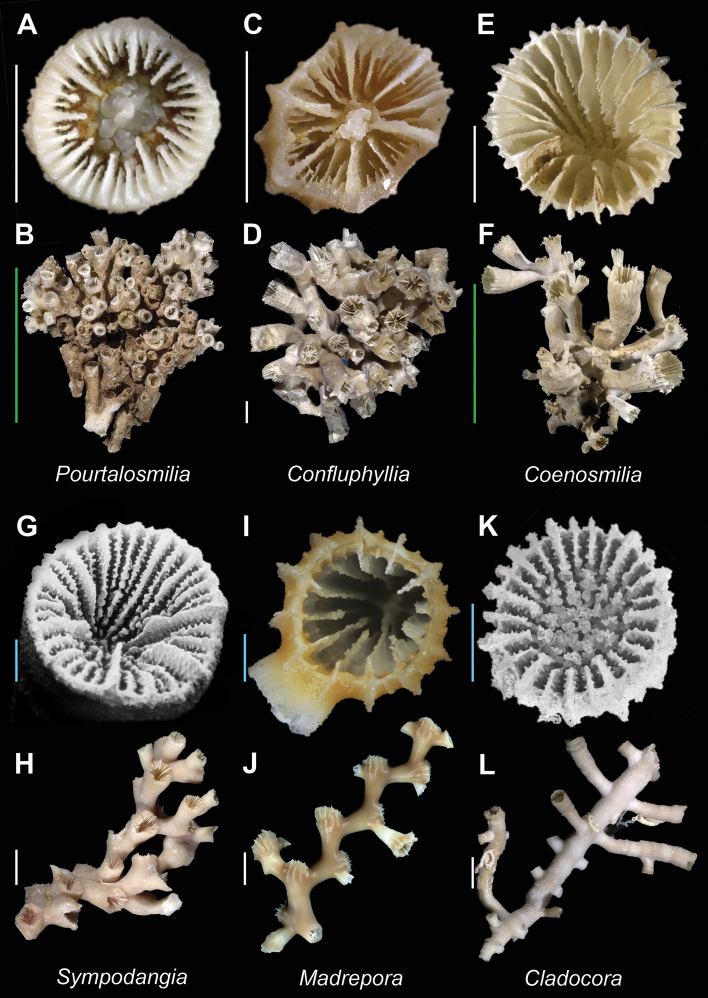
***Pourtalosmilia anthophyllite*sA** (USNM 1174947) and **B** (USNM 117494): Calicular and colony view respectively; ***Confluphyllia juncta***(USNM 97316, paratype) **C** and **D** Calicular and colony view respectively; ***Coenosmilia arbuscula***(USNM 97312) **E** and **F** Calicular and colony view respectively; ***Sympodangia albatrossi*** (USNM 97308, holotype) **G** (SEM) and **H** Calicular and colony view respectively; ***Madrepora oculata***(MNHN uncatalogued, Halipro 2 stn. BT104) **I** and **J** Calicular and colony view respectively; *Cladocora debilis* **K** (USNM 10452, SEM) and **L** (USNM 62351): Calicular and colony view respectively. Scale bars: blue = 1 mm; white = 5 mm; green = 50 mm. Bold face indicates type species for the genus.

**Plate 4.**
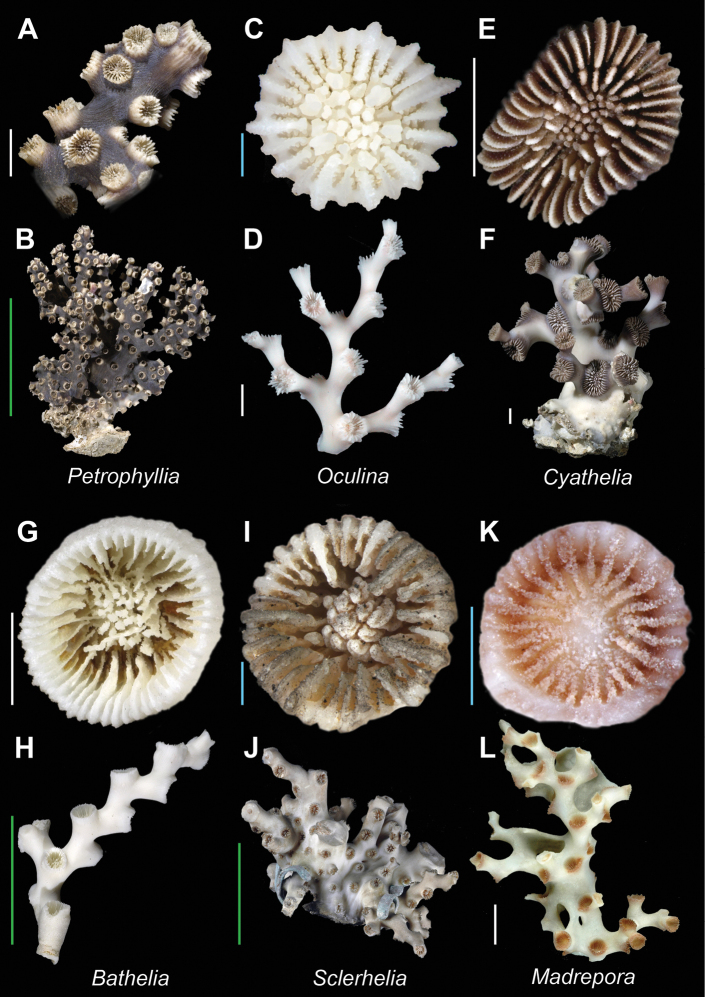
*Petrophyllia rediviva* (USNM 82696) **A** and **B** Distal branch and colony view respectively; *Oculina virgosa* (MNHN uncatalogued, SMIB 5 stn. DW101) **C** and **D** Calicular and colony view respectively; ***Cyathelia axillaris***(USNM 92665)**E** and **F** Calicular and colony view respectively; ***Bathelia candid*a**(USNM 47512) **G** and **H** Calicular and colony view respectively; ***Sclerhelia hirtella***(MNHN Michellin collection) **I** and **J** Calicular and colony view respectively; *Madrepora minutiseptum* (MNHN uncatalogued, SMIB 5 stn. DW101) **K** and **L** Calicular and colony view respectively. Scale bars: blue = 1 mm; white = 5 mm; green = 50 mm. Bold face indicates type species for the genus.

**Plate 5.**
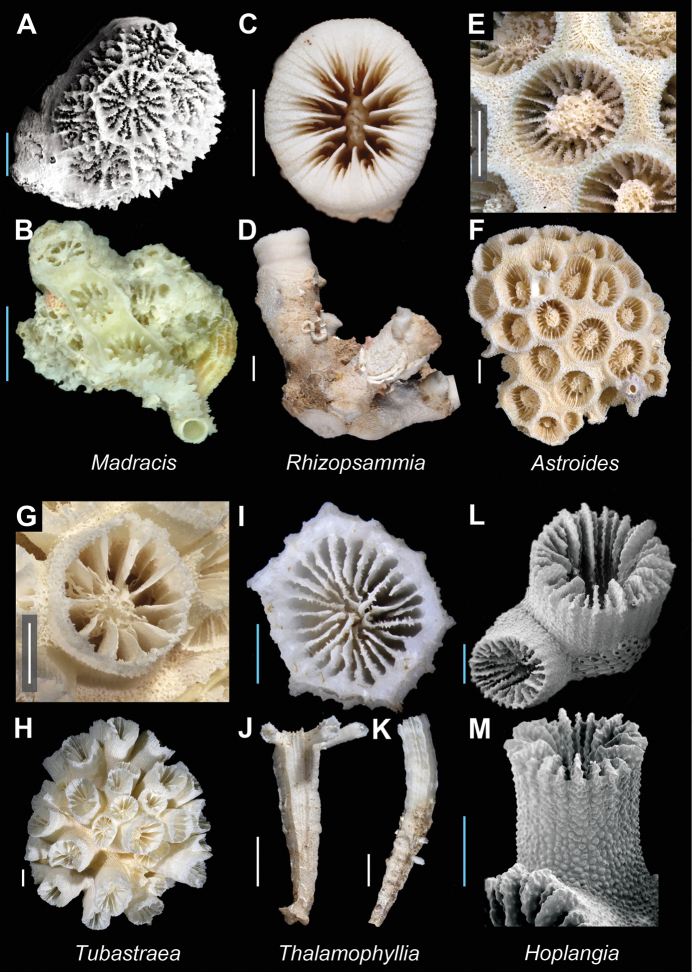
*Madracis pharensis* **A** (USNM 96676, SEM): Colony view; *Madracis* sp. **B** (MNHN uncatalogued, New Caledonia): Colony view; *Rhizopsammia* sp. (USNM uncatalogued, Bathus 4 stn. DW 941) **C** and **D** Calicular and colony view respectively; ***Astroides calycularis***(USNM 78767) **E** and **F **Calice detail and colony view respectively; ***Tubastraea coccine*a**(USNM 46973) **G** and **H** Calicular and colony view respectively; *Thalamophyllia tenuescens* **I** (MNHN uncatalogued, Bathus 3 stn. CH802), **J **and **K **(Norfolk 2 stn. 2095): Calicular and corallum views respectively; ***Hoplangia durotri*x**(AU 6097) **L **(SEM) and **M** (SEM): Calicular and lateral view respectively. Scale bars: blue = 1 mm; white = 5 mm; green = 50 mm. Bold face indicates type species for the genus.

**Plate 6.**
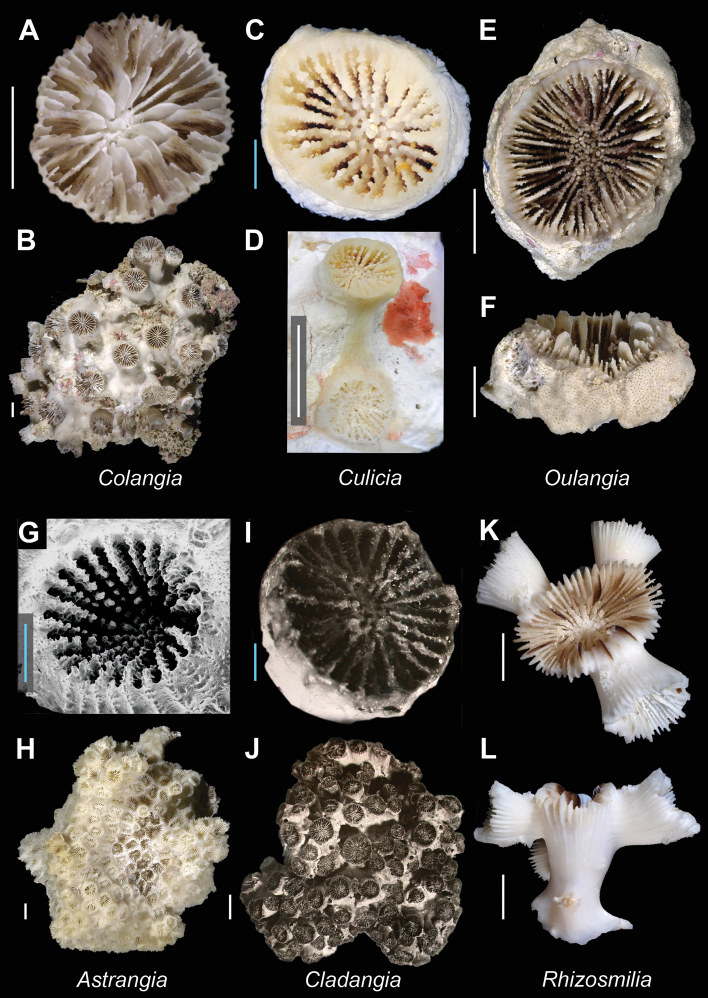
***Colangia immersa*** (USNM 73917) **A** and **B** Calicular and colony view respectively; ***Culicia stellat*a**(MNHN uncatalogued, New Caledonia) **C** and **D** Calicular and stolon connection view respectively; *Oulangia bradleyi* (USNM 92371)**E** and **F** Calicular and lateral view respectively; *Astrangia danae* (USNM 78507, SEM) **G** Calicular view; ***Astrangia poculata***(USNM 80350, neotype) **G** and **H** and colony view respectively; ***Cladangia exust*a** (YPM 1359, syntype ?) **I** and **J** Calicular and colony view respectively; *Rhizosmilia sagamiensis* (USNM uncatalogued, Norfolk 2 stn. 2124) **K** and **L** Calicular and lateral view respectively. Scale bars: blue = 1 mm; white = 5 mm. Bold face indicates type species for the genus.

**Plate 7.**
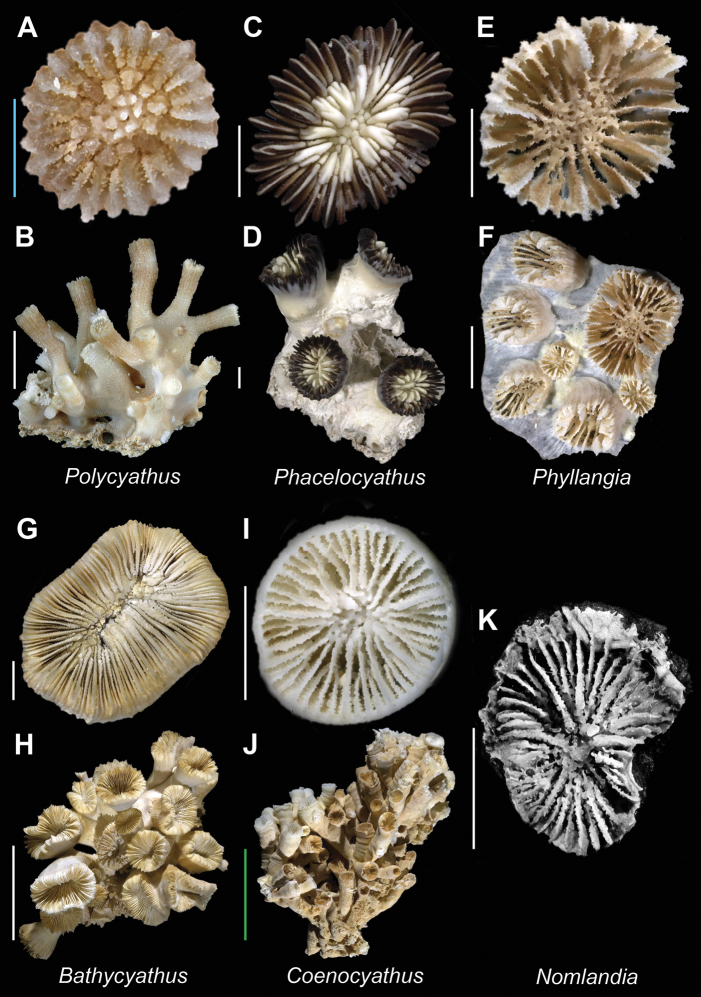
*Polycyathus* sp. (MNHN uncatalogued, Beryx 11 stn. DW11) **A** and **B** Calicular and colony view respectively; ***Phacelocyathus flos***(USNM 46077)**C** and **D** Calicular and colony view respectively; ***Phyllangia americana***(USNM 80881)**E** and **F** Calicular and colony view respectively; ***Bathycyathus chilensis***(USNM 100711) **G** and **H** Calicular and colony view respectively; *Coenocyathus anthophyllites* (USNM 48694)**I** and **J** Calicular and colony view respectively; ***Nomlandia californica***(SBMNH 35560, holotype)**K** Calicular view (after Cairns 1994). Scale bars: blue = 1 mm; white = 5 mm; green = 50 mm. Bold face indicates type species for the genus.

**Plate 8.**
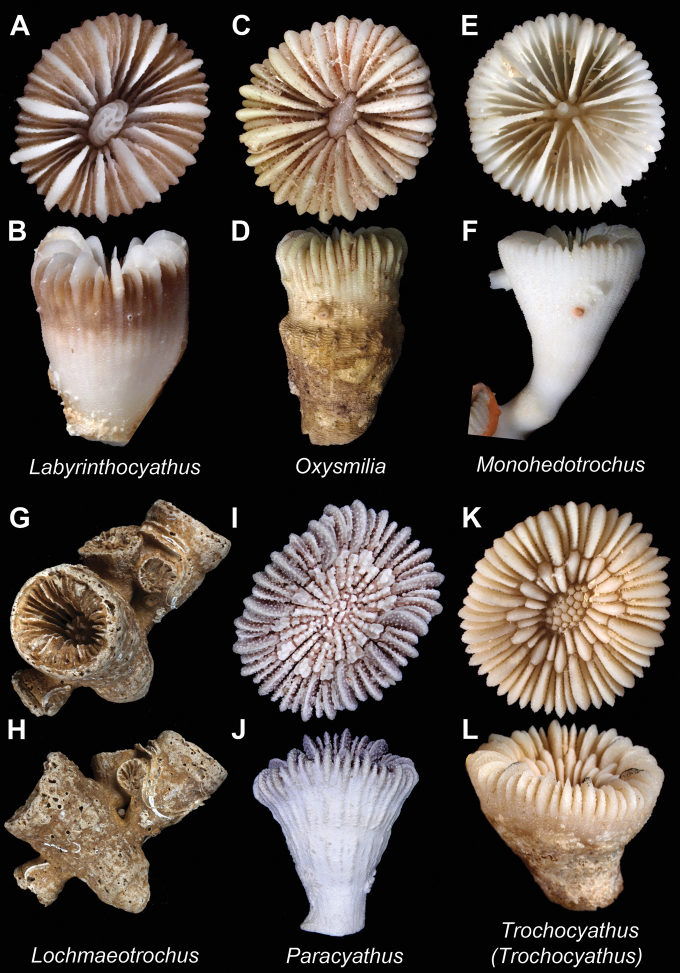
*Labyrinthocyathus limatulus* (USNM uncatalogued, Bathus 4 stn. DW 936)**A** and **B** Calicular and lateral view respectively; *Oxysmilia corrugata* (USNM uncatalogued, Norfolk 2 stn. DW2125)**C **and **D** Calicular and lateral view respectively; *Monohedotrochus circularis* (USNM uncatalogued, Norfolk 2 stn. DW2124)**E** and **F** Calicular and lateral view respectively; ***Lochmaeotrochus oculeus***(USNM uncatalogued, Musorstom 6 stn. DW394)**G** and **H** Calicular and “aggregation” view respectively; *Paracyathus* sp. (MNHN uncatalogued, Ebisco stn. DW2555) **I** and **J** Calicular and lateral view respectively; *Trochocyathus efateensis* (USNM uncatalogued, Bathus 4 stn. DW818) **K** and **L** Calicular and lateral view respectively. Scale bars represent 5 mm. Bold face indicates type species for the genus.

**Plate 9.**
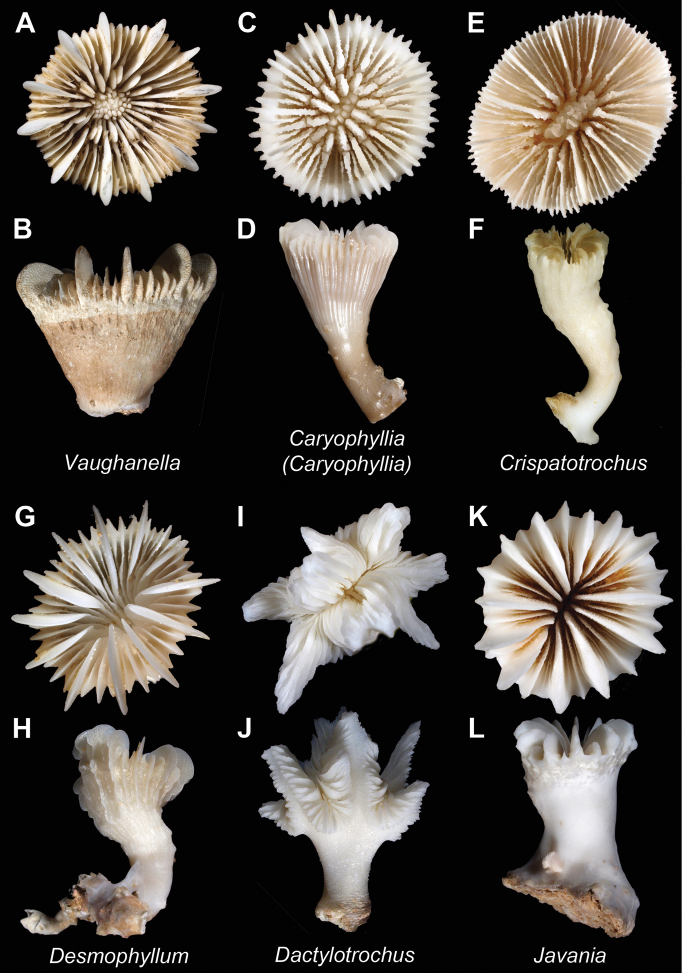
*Vaughanella concinna* (USNM uncatalogued, Norfolk 2 stn. DW2070) **A** and **B** Calicular and lateral view respectively; *Caryophyllia (Caryophyllia) diomedeae* (USNM uncatalogued, Norfolk 2 stn. DW2086) **C** and **D** Calicular and lateral view respectively; *Crispatotrochus rubescens* (USNM uncatalogued, Bathus 3 stn. CP833)**E** and **F** Calicular and lateral view respectively; ***Desmophyllum dianthus***(USNM uncatalogued, Halipro 1 stn. CP877) **G** and **H** Calicular and lateral view respectively; ***Dactylotrochus cervicorni*s**(USNM uncatalogued, SMIB 10 stn. DW208) **I** and **J** Calicular and lateral view respectively; ***Javania insigni*s**(USNM uncatalogued, Norfolk 2 stn. DW2023) **K** and **L** Calicular and lateral view respectively. Scale bars represent 5 mm. Bold face indicates type species for the genus.

**Plate 10.**
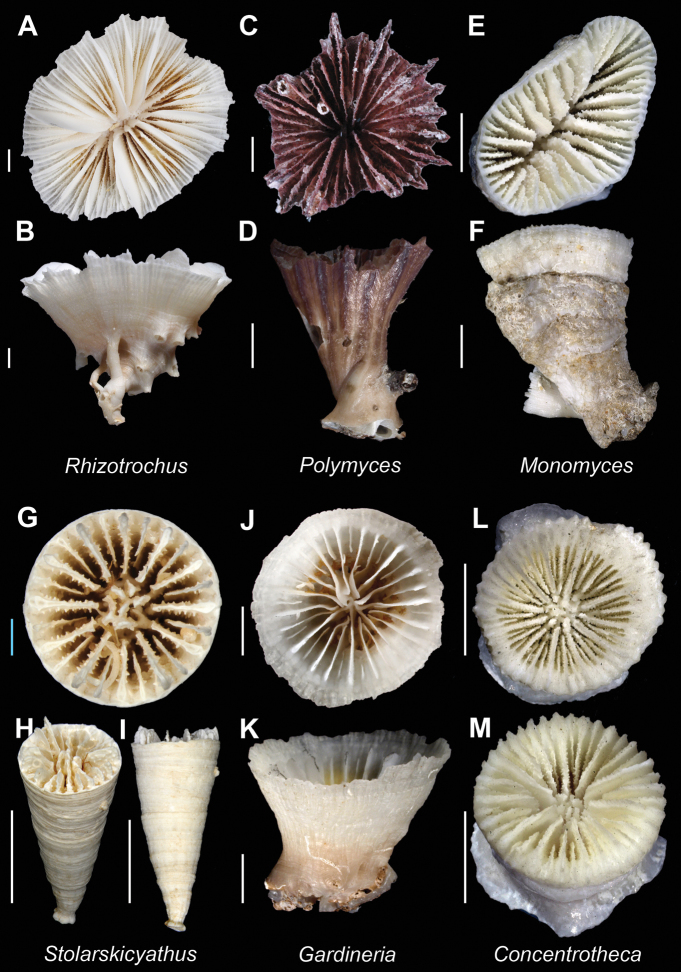
***Rhizotrochus typus***(USNM uncatalogued, Norfolk 2 stn. DW2024)**A** and **B** Calicular and lateral view respectively; *Polymyces wellsi* **C** (MNHN uncatalogued, Ebisco stn. DW2618) and **D **(MNHN uncatalogued, New Caledonia): Calicular and lateral view respectively; ***Monomyces pygmaea***(USNM 48561) **E** and **F** Calicular and lateral view respectively; ***Stolarskicyathus pocilliformis***(MNHN uncatalogued, Ebisco stn. DW2573) **G, H,** and **I** Calicular, oblique, and lateral view respectively; ***Gardineria hawaiiensis***(USNM uncatalogued, Norfolk 2 stn. DW2086) **J** and **K** Calicular and lateral view respectively; ***Concentrotheca laevigat*a**(USNM 80748) **L** and **M** Calicular and oblique view respectively. Scale bars: blue = 1 mm; white = 5 mm. Bold face indicates type species for the genus.

**Plate 11.**
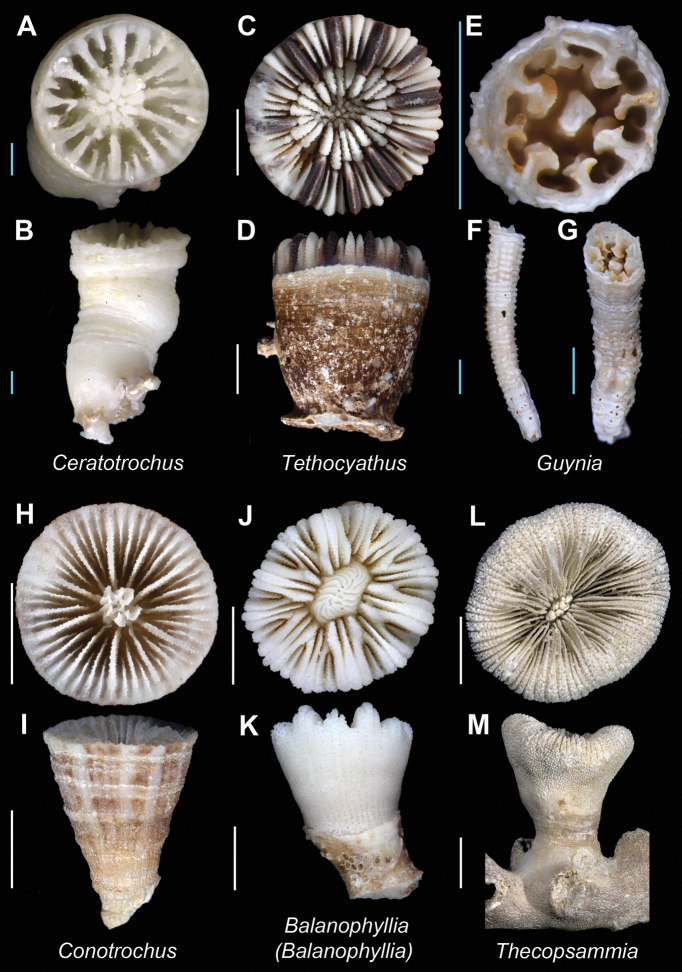
*Ceratotrochus magnaghii* (USNM 48780) **A** and **B** Calicular and lateral view respectively; *Tethocyathus virgatus* (USNM uncatalogued, SMIB 10 stn. DW205) **C** and **D** Calicular and lateral view respectively; ***Guynia annulata***(MNHN uncatalogued, Biogeocal stn. DW253) **E, F** and **G** Calicular, lateral, and oblique view respectively; *Conotrochus funicolumna* (USNM uncatalogued, Bathus 4 stn. CP967) **H** and **I** Calicular and lateral view respectively; *Balanophyllia (Balanophyllia) laysanensis* (MNHN uncatalogued, Musorstom 6 stn. DW407) **J** and **K** Calicular and lateral view respectively; ***Thecopsammia sociali*s**(USNM 61828) **L** and **M** Calicular and lateral view respectively. Scale bars: blue = 1 mm; white = 5 mm. Bold face indicates type species for the genus.

**Plate 12.**
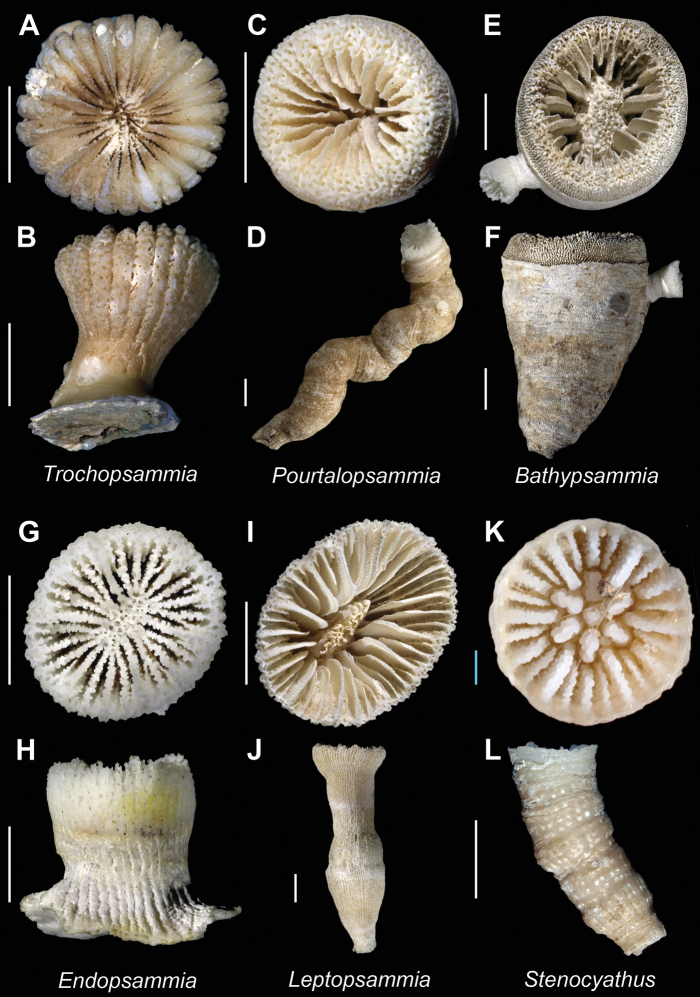
***Trochopsammia infundibulum***(USNM 46722)**A** and **B** Calicular and lateral view respectively; ***Pourtalopsammia togat*a**(USNM 91792) **C** and **D** Calicular and lateral view respectively; ***Bathypsammia tintinnabulum*** (USNM 14569)**E** and **F** Calicular and lateral view respectively; ***Endopsammia philippensi*s**(USNM 83006) **G** and **H** Calicular and lateral view respectively; ***Leptopsammia stokesian*a**(USNM 78603) **I** and **J** Calicular and lateral view respectively; ***Stenocyathus vermiformi*s**(USNM uncatalogued, Norfolk 2 stn. ?) **K** and **L** Calicular and lateral view respectively. Scale bars: blue = 1 mm; white = 5 mm. Bold face indicates type species for the genus.

**Plate 13.**
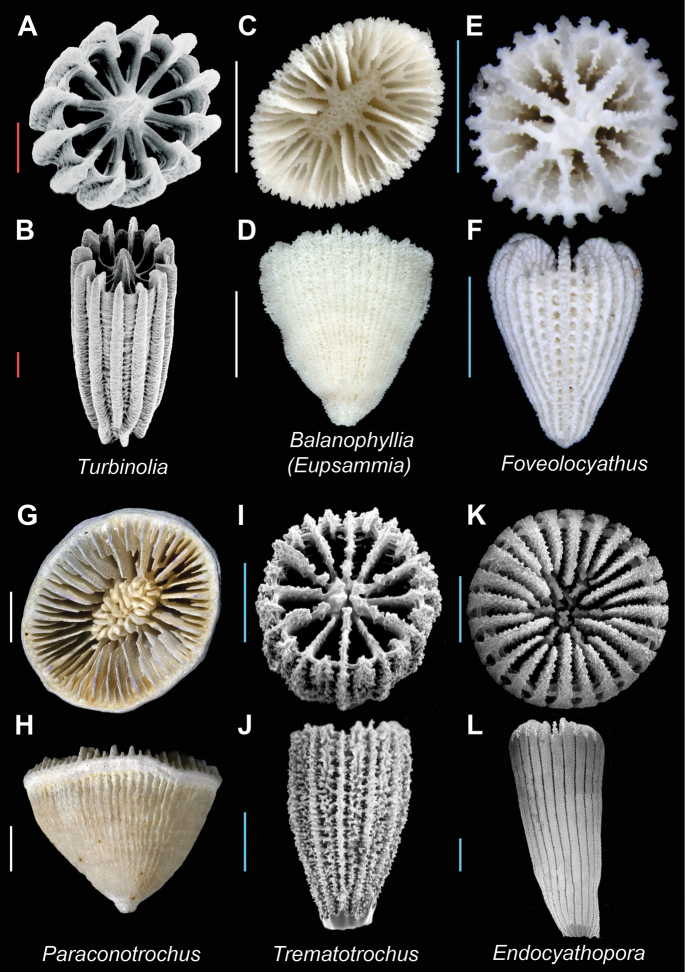
*Turbinolia stephensoni* (USNM 80014) **A** (SEM) and **B** (SEM): Calicular and oblique view respectively; *Balanophyllia (Eupsammia) carinata* (MNHN uncatalogued, Chalcal stn. D22) **C** and **D** Calicular and lateral view respectively; *Foveolocyathus parkeri* (MNHN uncatalogued, Musorstom 5 stn. 280)**E** and **F** Calicular and lateral view respectively; ***Paraconotrochus zeidler*i**(USNM 85677, paratype) **G **and **H** Calicular and lateral view respectively; *Trematotrochus corbicula* (USNM 46477) **I** (SEM) and **J** (SEM): Calicular and lateral view respectively; ***Endocyathopora laticostat*a** (USNM 81894) **K **(SEM) and **L** (SEM): Calicular and lateral view respectively. Scale bars: red = 0.25 mm; blue = 1 mm; white = 5 mm; green = 50 mm. Bold face indicates type species for the genus.

**Plate 14.**
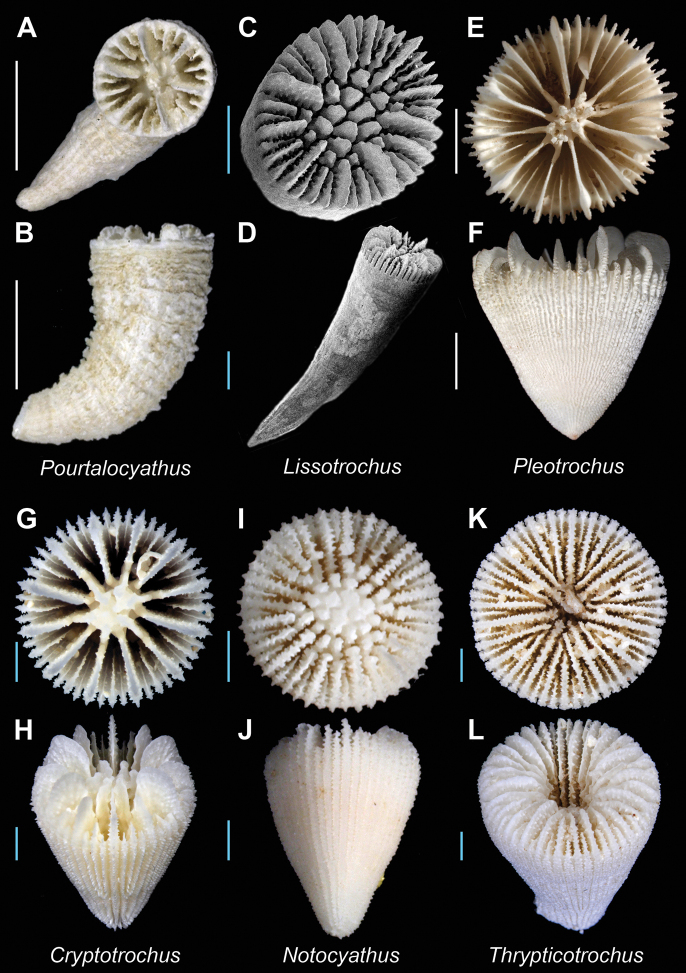
***Pourtalocyathus hispidus***(USNM 61928)**A** and **B** Calicular and lateral view respectively; ***Lissotrochus curvatus***(AM G16745) **C** (SEM) and **D** (SEM): Calicular and oblique view respectively; ***Pleotrochus venustus***(USNM uncatalogued, Norfolk 2 stn. DW 2104) **E** and **F** Calicular and lateral view respectively; *Cryptotrochus* sp. (MNHN uncatalogued, Ebisco stn. DW2603) **G** and **H** Calicular and oblique view respectively; *Notocyathus venustus* (USNM uncatalogued, Bathus 4 stn. DW 958) **I** and **J** Calicular and lateral view respectively; ***Thrypticotrochus petterdi***(MNHN uncatalogued, Ebisco stn. DW2561) **K** and **L** Calicular and oblique view respectively. Scale bars: blue = 1 mm; white = 5 mm. Bold face indicates type species for the genus.

**Plate 15.**
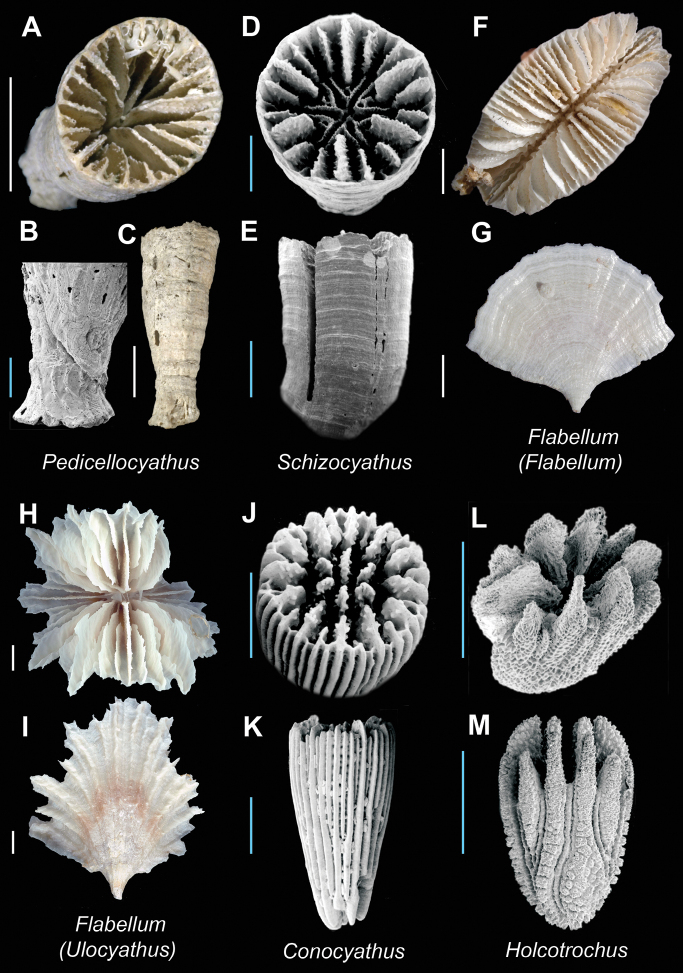
***Pedicellocyathus keyesi***(USNM 94268, paratype) **A, B** (SEM), and **C** Calicular, pedicel detail, and lateral view respectively; ***Schizocyathus fissili*s**(USNM 61747) **D** (SEM) and **E** (SEM): Calicular and lateral view respectively; *Flabellum (Flabellum) politum* (USNM uncatalogued, Bathus 4 stn. DW933) **F** and **G** Calicular and lateral view respectively; *Flabellum (Ulocyathus) messum* (MNHN uncatalogued, Bathus 1 stn. DW661) **H** and **I** Calicular and lateral view respectively; *Conocyathus zelandiae* (USNM 85713) **J** (SEM) and **K** (SEM): Oblique and lateral view respectively; ***Holcotrochus scriptus*** (USNM 85687) **L** (SEM) and **M** (SEM): Oblique and lateral view respectively. Scale bars: blue = 1 mm; white = 5 mm. Bold face indicates type species for the genus.

**Plate 16.**
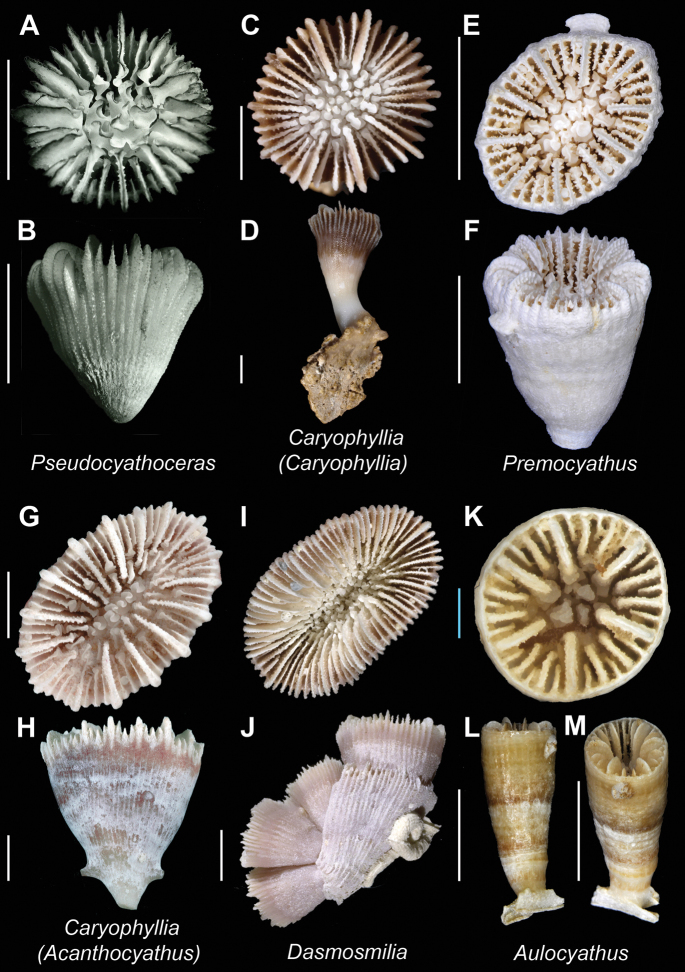
***Pseudocyathoceras avis***(USNM 46962, holotype)**A** and **B** Calicular and lateral view respectively; *Caryophyllia (Caryophyllia) quadragenaria* (USNM uncatalogued, PrFO, New Caledonia) **C** and **D** Calicular and oblique view respectively; ***Premocyathus dentiformis***(MNHN uncatalogued, Ebisco stn. DW2573) **E** and **F** Calicular and oblique view respectively; ***Caryophyllia (Acanthocyathus) gra*yi**(MNHN uncatalogued, Ebisco stn. DW2559) **G** and **H** Calicular and lateral view respectively; ***Dasmosmilia lyman*i**(USNM 82997) **I** and **J** Calicular and lateral (aggregation) view respectively; ***Aulocyathus juvenescens***(MNHN uncatalogued, Lifou 2000 stn. DW37) **K, L,** and **M** Calicular, lateral, and oblique view respectively. Scale bars: blue = 1 mm; white = 5 mm. Bold face indicates type species for the genus.

**Plate 17.**
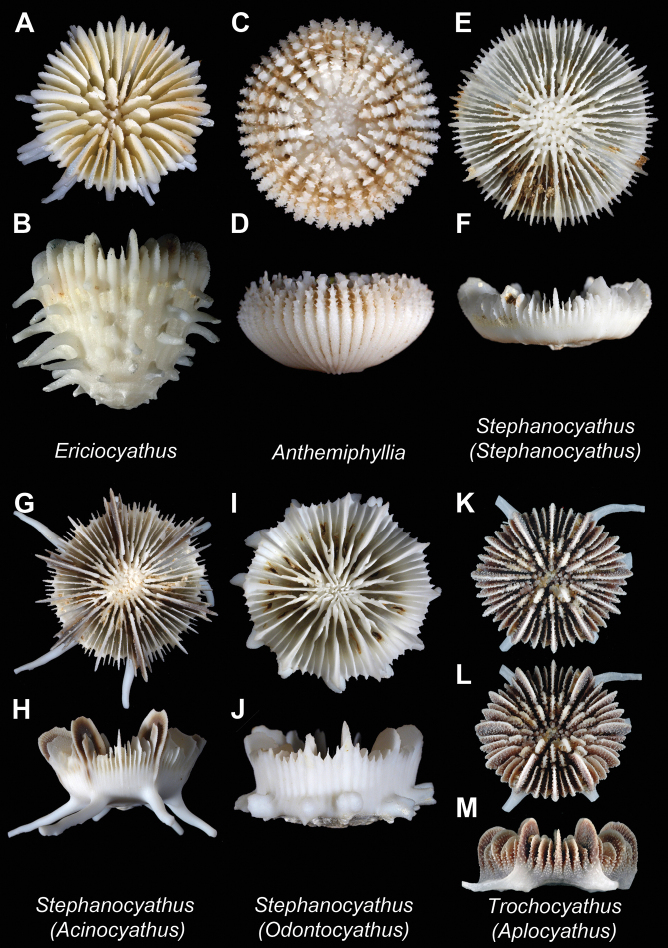
***Ericiocyathus echinatus***(USNM 97169, holotype)**A** and **B** Calicular and lateral view respectively; *Anthemiphyllia patera costata* (USNM uncatalogued, Norfolk 2 stn. 2066) **C** and **D** Calicular and lateral view respectively; *Stephanocyathus (Stephanocyathus) regius* (USNM uncatalogued, Bathus 3 stn. 858) **E** and **F** Calicular and lateral view respectively; ***Stephanocyathus (Acinocyathus) spinig*er**(USNM uncatalogued, Bathus 3 stn. CP877) **G** and **H** Calicular and lateral view respectively; ***Stephanocyathus (Odontocyathus) coronat*us** (USNM uncatalogued, Bathus 4 stn. CP950) **I** and **J** Calicular and lateral view respectively; *Trochocyathus (Aplocyathus) brevispina* (MNHN uncatalogued, Musorstom 8 stn. DW960) **K, L,** and **M** Calicular, oblique, and lateral view respectively. Scale bars represent 5 mm. Bold face indicates type species for the genus.

**Plate 18.**
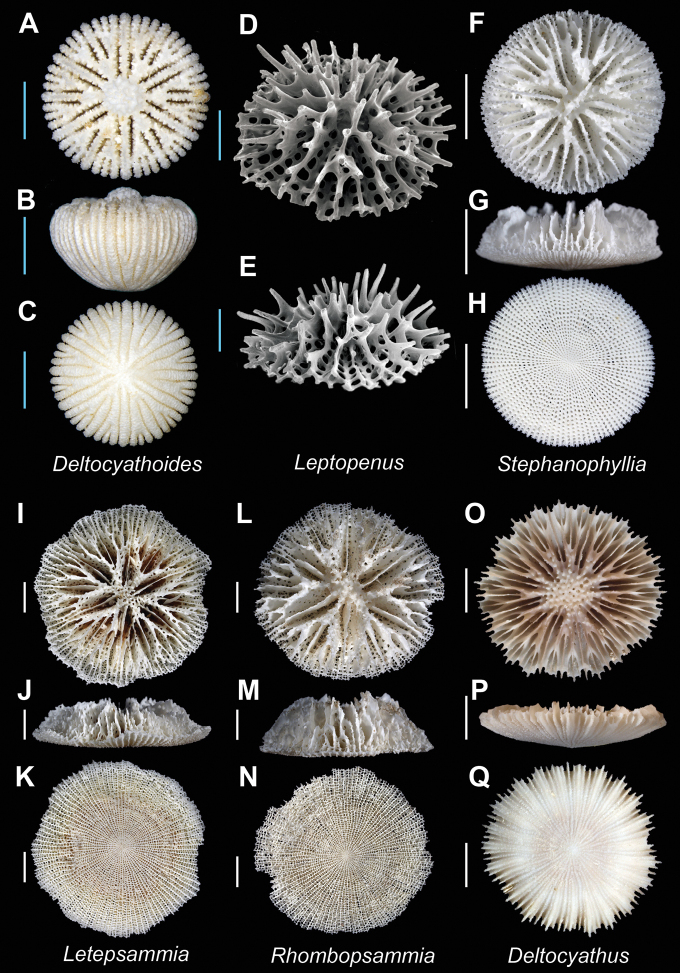
***Deltocyathoides orientalis***(MNHN uncatalogued, Bathus 3 stn. DW829) **A, B** and **C  **Calicular, lateral, and base view respectively; ***Leptopenus discu*s**(SIO Co-1271) **D** (SEM) and **E** (SEM): Oblique views respectively; *Stephanophyllia complicata* (USNM uncatalogued, New Caledonia) **F, G,  **and **H** Calicular, lateral, and base view respectively; ***Letepsammia formosissim*a**(USNM uncatalogued, Norfolk 2 stn. DW2032) **I, J,** and **K** Calicular, lateral, and base view respectively; *Rhombopsammia niphada* (USNM uncatalogued, Norfolk 2 stn. DW2069) **L, M,** and **N** Calicular, lateral, and base view respectively; *Deltocyathus rotulus* (MNHN-Scl.2008-0004) **O, P,** and **Q** Calicular, lateral, and base view respectively. Scale bars: blue = 1 mm; white = 5 mm. Bold face indicates type species for the genus.

**Plate 19.**
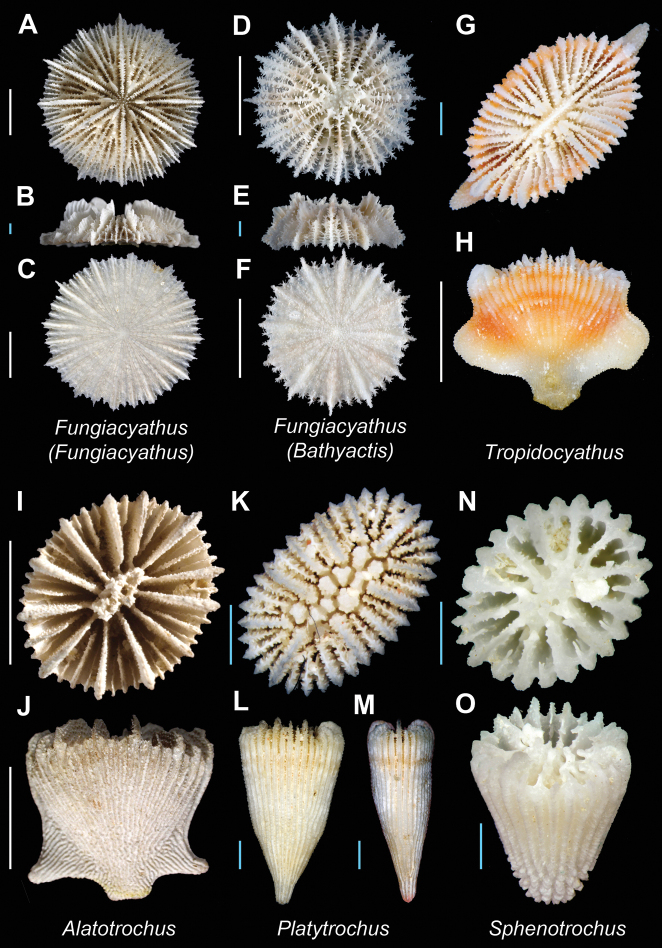
*Fungiacyathus (Fungiacyathus) paliferus* (USNM uncatalogued, Bathus 3 stn. DW 887) **A, B **and **C** Calicular, lateral, and base view respectively; *Fungiacyathus (Bathyactis) variegatus* (MNHN uncatalogued, Lagoon NO stn. DC933) **D, E,** and **F** Calicular, lateral, and base view respectively; ***Tropidocyathus lessoni***(MNHN uncatalogued, Musorstom 8 stn. DW1105) **G** and **H** Calicular and lateral view respectively; ***Alatotrochus rubescen*s**(USNM uncatalogued, Bathus 4 stn. DW 908) **I** and **J** Calicular and lateral view respectively; *Platytrochus hastatus* (MNHN uncatalogued, Ebisco stn. DW2559) **K, L,** and **M** Calicular and lateral (GCD and LCD) views respectively; *Sphenotrochus hancocki* (MNHN uncatalogued, Ebisco stn. DW2617) **N** and **O** Calicular and oblique view respectively. Scale bars: blue = 1 mm; white = 5 mm. Bold face indicates type species for the genus.

**Plate 20.**
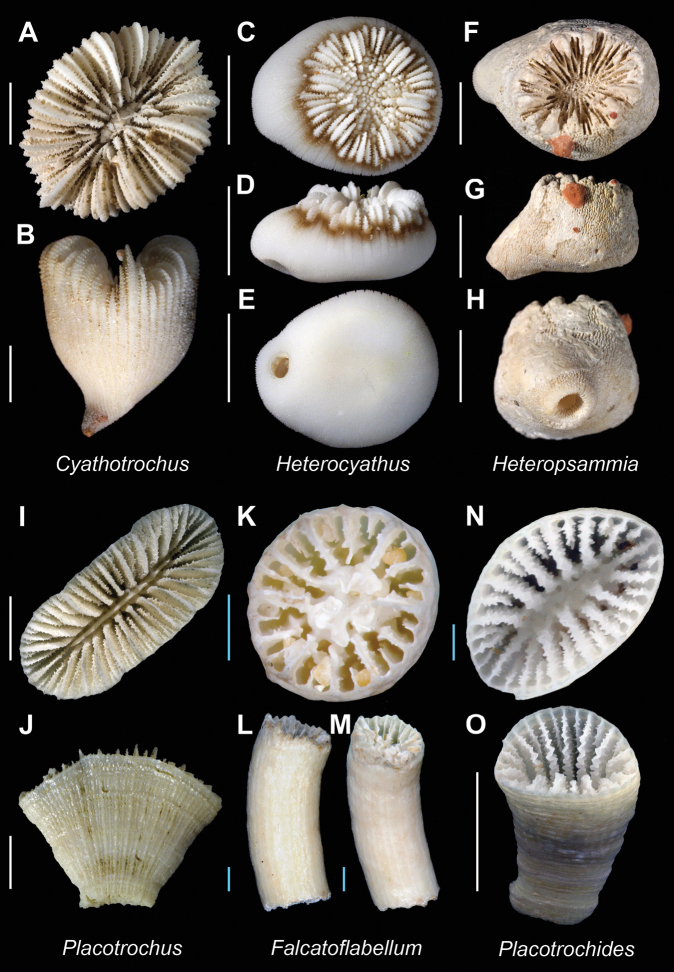
***Cyathotrochus pileus***(USNM uncatalogued, Bathus 3 stn. CP833) **A** and **B** Calicular and lateral view respectively; ***Heterocyathus aequicostatus***(USNM uncatalogued, Bathus 4 stn. DW933) **C, D,** and **E** Calicular, lateral, and base view respectively; ***Heteropsammia cochlea***(USNM uncatalogued, Bathus 3 stn. DW894)**F, G,** and **H** Calicular, lateral, and base view respectively; ***Placotrochus laevi*s**(USNM 81989) **I** and **J** Calicular and lateral view respectively; ***Falcatoflabellum raoulensi*s**(MNHN uncatalogued, Ebisco stn. DW2603) K, L, and **M** Calicular, lateral, and oblique view respectively; ***Placotrochides scaphul*a**(MNHN uncatalogued, Chalcal stn. DW75) **N** and **O** Calicular and oblique view respectively. Scale bars: blue = 1 mm; white = 5 mm. Bold face indicates type species for the genus.

**Plate 21.**
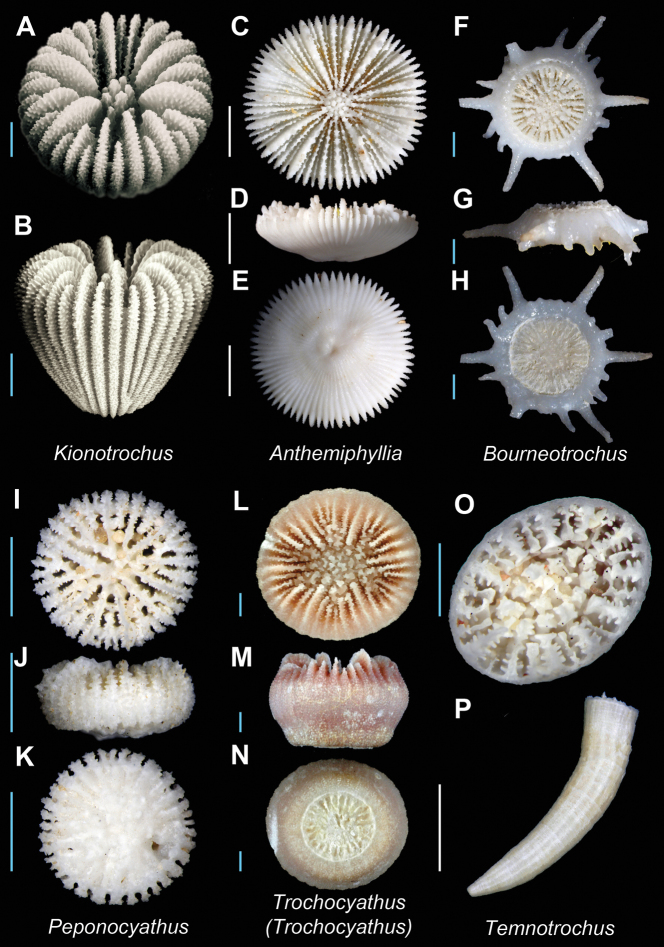
***Kionotrochus suter*i**(NZOI F915) **A** (SEM) and **B** (SEM): Calicular and lateral view respectively; *Anthemiphyllia dentata* (USNM uncatalogued, Bathus 4 stn. DW914) **C, D,** and **E** Calicular, lateral, and base view respectively; ***Bourneotrochus stellulatus***(USNM uncatalogued, Bathus 3 stn. DW877) **F, G,** and **H** Calicular, lateral, and base view respectively; ***Peponocyathus folliculu*s**(MNHN uncatalogued, Norfolk 1 stn. DW1697) **I, J**, and **K** Calicular, lateral, and base view respectively; *Trochocyathus (Trochocyathus) discus* (MNHN uncatalogued, Biocal stn. DW46) **L, M,** and **N** Calicular, lateral, and base view respectively; ***Temnotrochus kermadecensi*s**(MNHN uncatalogued, Musorstom 5 stn. DW328) **O** and **P** Calicular and lateral view respectively. Scale bars: blue = 1 mm; white = 5 mm. Bold face indicates type species for the genus.

**Plate 22.**
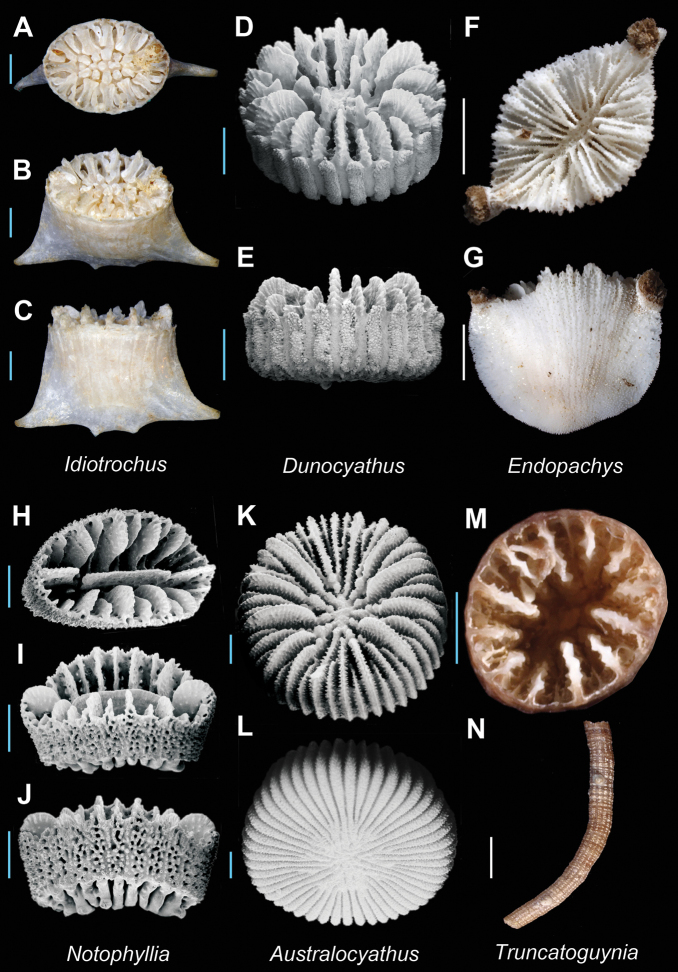
***Idiotrochus emarciatu*s**(MNHN uncatalogued, Ebisco stn. DW2632) **A, B,** and **C** Calicular, oblique, and lateral view respectively; ***Dunocyathus parasiticu*s**(USNM 85697) **D** (SEM) and **E** (SEM): Oblique and lateral view respectively; *Endopachys grayi* (USNM uncatalogued, Norfolk 2 stn. DW2158)**G** and **H** Calicular and lateral view respectively; *Notophyllia recta* (USNM 85752) **H, I,** and **J** Calicular, oblique, and lateral view respectively; ***Australocyathus vincentinus***(USNM 85699) **K** and **L** Oblique views respectively; ***Truncatoguynia irregularis***(USNM uncatalogued, Norfolk 2 stn. DW2117) **M** and **N** Calicular and lateral view respectively. Scale bars: blue = 1 mm; white = 5 mm. Bold face indicates type species for the genus.

**Plate 23.**
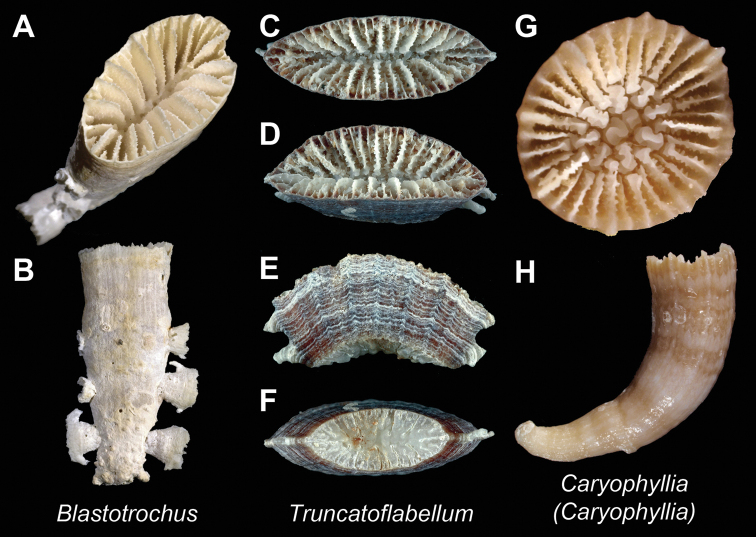
***Blastotrochus nutri*x**(USNM 97553)– **A** and **B** Calicular and lateral view respectively; *Truncatoflabellum* sp. (MNHN uncatalogued, Concalis stn. DW2934) **C, D, E,** and **F** Calicular, oblique, lateral, and basal scar view respectively; *Caryophyllia (Caryophyllia) abrupta* (MNHN-Scl.2009-0067) **G **and **H** Calicular and lateral view respectively. Scale bars: blue = 1 mm; white = 5 mm. Bold face indicates type species for the genus.

**Plate 24.**
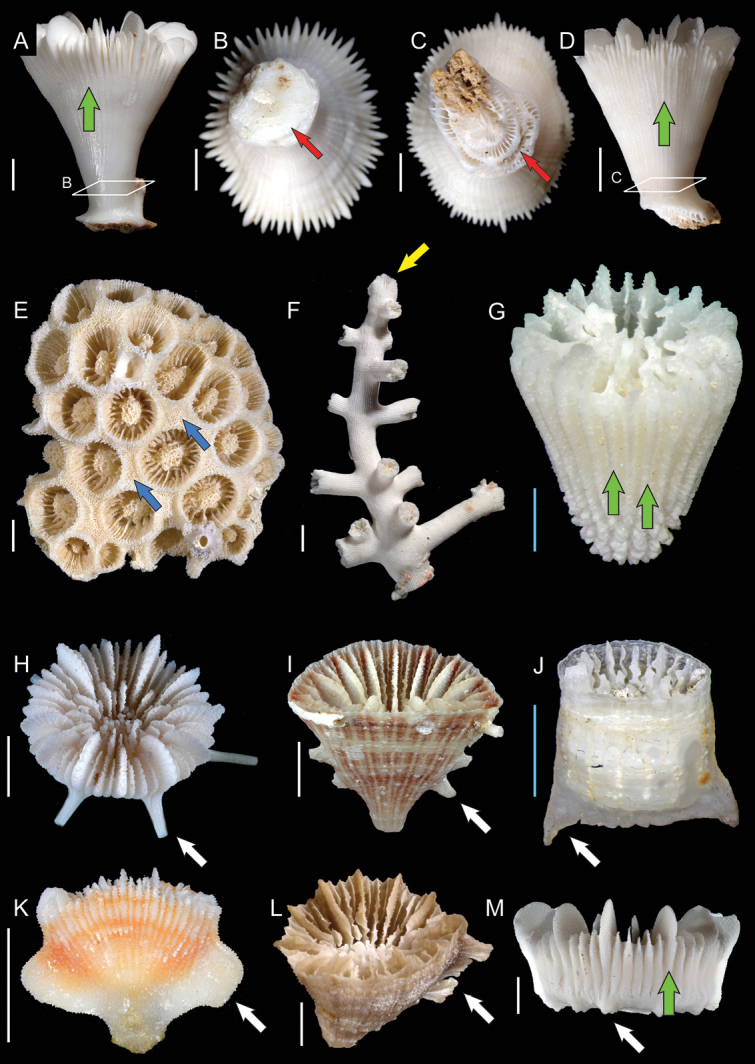
**A** and **B** *Caryophyllia ralphae* (MNHN-Scl.2009-0077, **A** lateral view and **B** pedicel section): Green and red arrows indicating costae and monocyclic base respectively **C** and **D** *Rhizosmilia robusta* (USNM uncatalogued, Norfolk 2 stn. DW2114 **C** pedicel section and **D** lateral view): Red and green arrows indicating polycyclic base and costae respectively **E** *Astroides calycularis* (USNM 78767, colony view): Blue arrows indicating coenosteum **F** *Dendrophyllia ijimai* (USNM uncatalogued, Bathus 4 stn. DW933, lateral colony view): Yellow arrow indicating the axial polyp **G** *Sphenotrochus hancocki* (MNHN uncatalogued, Ebisco stn. DW2617, oblique view): Green arrows indicating costae **H** *Trochocyathus hastatus* (MNHN uncatalogued, Ebisco stn. DW2497, oblique view): White arrow indicating costal spines **I ***Truncatoflabellum vigintifarium* (MNHN uncatalogued, Ebisco stn. DW2578, oblique view): White arrow indicating lateral edge spines **J** *Idiotrochus emarciatus* (MNHN uncatalogued, Ebisco stn. DW2632, lateral view): White arrow indicating lateral edge spines (fish tail) **K** *Tropidocyathus lessoni* (MNHN uncatalogued, Musorstom 8 stn. DW1105, lateral view): White arrow indicating alate edge crests **L** *Caryophyllia unicristata* (MNHN-Scl.2009-0094, oblique view): White arrow indicating very sinuous lateral crest **M ***Stephanocyathus weberianus* (MNHN uncatalogued, Musorstom 5 stn. DW313, lateral view): White and green arrows indicating tubercles and costae respectively; Scale bars: blue = 1 mm; white = 5 mm.

**Plate 25.**
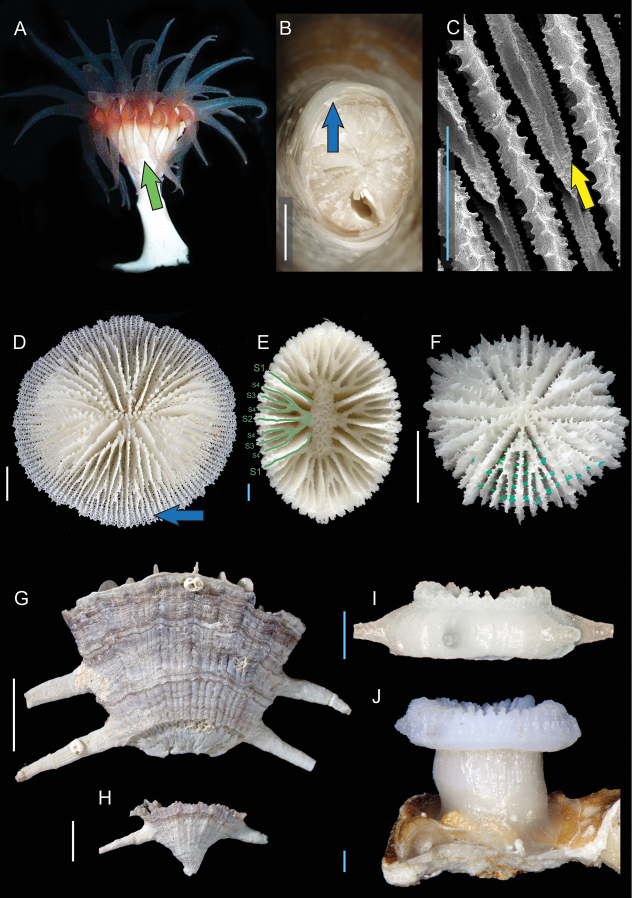
**A** Indeterminate Caryophylliina (lateral view of live specimen, Roatan, Honduras ~200 m deep): Green arrow indicating the edge zone **B** *Javania* sp. (USNM uncatalogued, Norfolk 2 stn. CH 2115, broken pedicel section): Blue arrow indicating tectura layers **C** *Leptoseris gardineri* (JCU uncatalogued, Australia, septal detail [SEM]): Yellow arrow indicating meniane **D** *Letepsammia franki* (MNHN uncatalogued, Musorstom 6 stn. CP464, oblique view): Blue arrow indicating marginal shelf **E** *Balanophyllia carinata* (MNHN uncatalogued, Chalcal stn. D22, calicular view): Green diagram indicating a complete septal system arranged in a Pourtalès Plan configuration **F** *Fungiacyathus* sp. (MNHN uncatalogued, Biocal stn. CP17, oblique view): Synapticular plates highlighted in green **G** and **H** *Truncatoflabellum candeanun* (CSIRO uncatalogued, SS102005 stn. 170-086, lateral views) **G** anthocyathus and **H** anthocaulus **I** and **J ***Bourneotrochus stellulatus* (MNHN uncatalogued, Musorstom 4 stn. DW162, lateral views) **I** anthocyathus and **J** a specimen undergoing transverse division. Scale bars: blue = 1 mm; white = 5 mm.

## Glossary

**Anthocaulus**: See Transverse Division.

**Anthocyathus**: See Transverse Division.

**Apozooxanthellate**: Species that have facultative symbiotic relationships with unicellular photosynthetic dinoflagellates (*Symbiodinium* spp.).

**Axial Corallite**: See Corallite.

**Axial Septal Margin**: See Septum.

**Azooxanthellate**: Species that do not have symbiotic relationships with unicellular photosynthetic dinoflagellates (*Symbiodinium* spp.).

**Base (Figure 1)**: The lower several millimeters of a solitary corallum, usually solid and composed of an accretion of thecal layers (a **monocyclic base**) **(e.g. Plate 24, Fig. B)**, but in some genera composed of concentric rings of partitioned chambers, called a **polycyclic base** **(e.g. Plate 24, Fig. C)** (Cairns 1978). The base usually expands basally as a thin layer facilitating adhesion to the substrate.

**Budding**: The process of asexual reproduction that adds new mouths (or polyps) to a corallum, often resulting in a colony. **Intratentacular budding** adds new polyps to the oral disc inside the ring of tentacles surrounds its mouth **(e.g. Plate 1, Fig. K–L)**. **Extratentacular budding** adds new polyps outside the ring of tentacles **(e.g. Plate 2, Fig. H)**. (The third form of asexual reproduction is **transverse division -** Cairns 1988b).

**Calice (Figure 1) (**pl. **Calices):** The skeletal analog of the polyp, cupping the polyp from below, and consisting of the septa, and, if present, the columella and pali **(e.g. Plate 1, Figs C, E, G; Plate 9, Figs A, C, E, G, I, K)**.

**Ceratoid Corallum**: See Solitary Corallum.

**Coenosteum**: The skeletal structure found between the individual corallites of a colonial corallum, including the costae, and various kinds of dissepiments; sometimes called **peritheca (e.g. Plate 24, Fig. E)**.

**Colonial Corallum**: See Corallum.

**Columella (Figure 1):** An axial structure of diverse shape and composition that projects from the center of a calice. If in the shape of a single lamella (called **lamellar**) **(e.g. Plate 20, Fig. I)**, if a maze of interconnected lamellae (**labyrinthiform**) **(e.g. Plate 8, Fig. A)**, if a set of twisted lamellae (**fascicular**) **(e.g. Plate 20, Figs C, E)**, if a simple rod (**styliform**) **(e.g. Plate 13, Fig. A)**, if a group of rods (**papillose**) **(e.g. Plate 8, Fig. K)**, if a fine porous mass (**spongy**) **(e.g. Plate 12, Fig. G)**, and if an irregular group of twisted elements (**trabecular**) **(e.g. Plate 16, Fig. K)**.

**Conical Corallum**: See Solitary Corallum.

**Corallite**: The vertical, usually cylindrical, structure produced by an individual polyp, consisting of endothecal dissepiments and the calice at the upper end **(e.g. Plate 1, Fig. F)**. If a corallite occurs at the tip of a colony’s branch, it is termed an **axial corallite (e.g. Plate 24, Fig. F)**.

**Corallum (Figure 1) (**pl. **Coralla)**: The aragonitic calcium carbonate skeleton of a scleractinian coral. If the coral has only one mouth (or calice), it is termed **solitary (e.g. Plate 10, Figs A–M; Plate 17, Figs A–M)**, if polystomatous (or more than one calice), then a **colonial (e.g. Plate 2, Fig. A–L; Plate 3, Figs A–L)**.

**Costae (Figure 1) (**sing. **Costa;** adj. **Costate):** Continuation of a septum on the outside of the corallite wall, often as a ridge or low linear mound **(e.g. Plate 24, Figs A, D, G, M)**.

**Crest**: See Edge Spine.

**Crown (Figure 1)**: See Palus.

**Cuneiform Corallum**: See Solitary Corallum.

**Cupolate Corallum**: See Solitary Corallum.

**Cycle**: See Septum.

**Cylindrical Corallum**: See Solitary Corallum.

**Discoidal Corallum**: See Solitary Corallum.

**Dissepiments**: Thin horizontal (**tabular dissepiments, e.g., Plate 2, Fig. K**) or blister-like plates that form within a corallite (**endothecal**) or beneath the coenosteum outside corallites (**exothecal**), which separate the polyp from the lower part of the corallum that it no longer occupies.

**Distomodeal Budding**: A mode of intratentacular budding in which two polyps (or calices) develop within the common tentacular ring (e.g., Plate 1, Figs **I–K**).

**Edge Spine/Crest/Spur**: The external thecal edges of a solitary coral, those associated with the principal septa, sometimes bears a low thin crest, or a series of hollow spines. If the crest is limited to the basal portion of the corallum and project outward in the shape of a fish tail, they may be called spurs **(e.g. Plate 24, Figs H–M)**.

**Edge Zone**: The fold of the polyp body that extends over the edge of the theca **(e.g. Plate 25, Fig. A)**.

**Endothecal**: See Dissepiments.

**Epitheca**: Thin, external, smooth or wrinkled, non-trabecular sheath surrounding individual corallites, formed by centripetal (inward) growth **(e.g. Plate 10, Fig. I, K; Plate 11, Figs D, I; Plate 12, Fig. F)**. **Tectura** is very similar in outward appearance by originates by centrifugal (outward) growth **(e.g. Plate 25, Fig. B)** (Stolarski 1995).

**Exothecal**: See Dissepiments.

**Extratentacular Budding**: See Budding.

**Fascicular Columella**: See Columella.

**Free**: An unattached corallum **(e.g. Plate 14, Figs A–L; Plate 17, Figs A–M; Plate 18, Figs A–Q)**.

**Globular Corallum**: See Solitary Corallum.

**Imperforate Theca**: See Theca.

**Intratentacular Budding**: See Budding.

**Labyrinthiform Columella**: See Columella.

**Lamellar Columella**: See Columella.

**Marginal Shelf**: A low rim encircling a solitary corallum composed of greatly reduced septa and costae, or costal spines (Cairns 1989) **(e.g. Plate 25, Fig. D)**.

**Menianes**: Short ledge-like features on septal faces formed by aligned lateral extensions of trabeculae **(e.g. Plate 25, Fig. C)**.

**Monocyclic Base**: See Base.

**Monostomaeous/Monostomatous**: A single-mouthed corallum, i.e., a solitary form **(e.g. Plate 8, Figs A–F)**.

**Normal Arrangement of Septa**: Arrangement of septa within a calice in which the septa are independent and all aligned with the center of the calice. (See Pourtalès Plan) **(e.g. Plate 8, Fig. E; Plate 9, Fig. E)**.

**Paliform Lobes**: Small, flattened lobes on the axial septal edge of various cycles, often more than one per septum, and part of the septum to which they are attached **(e.g., Plate 17, Fig. I)**.

**Palus (Figure 1)** (pl. **Pali**): Small flattened lobes on the axial septal edge of various cycles, always one per septum, and not part of the septum to which it is attached but ontogenetically different. Groups of pali occurring on the same cycle of septa and thus stand at the same distance from the center of the calice are called **crowns** of pali **(e.g. Figure 1)**.

**Papillose Columella**: See Columella.

**Parricidal Budding**: A mode of intratentacular budding in which new polyps are generated from the inner surface of a fragment of a parent corallum that has longitudinally split apart **(e.g. Plate 16, Fig. J, L–M)**.

**Pedicel (Figure 1)**: The stem-like region of a solitary coral just above the base and below the calicular surface.

**Perforate Theca**: See Theca.

**Peritheca**: See Coenosteum.

**Polycyclic Base:** See Base.

**Polystomaeous**/Polystomatous: See Corallum.

**Pourtalès Plan**: A form of septal arrangement in which the axial edges of pairs of higher cycle septa bend in front of and unite before their adjacent lower cycle septum **(e.g. Plate 25, Fig. E)**. See Cairns (1994: fig. 2).

**Reptoid Budding**: A type of extratentacular budding in which polyps are asexually generated from a thin, reticulate, encrusting ribbon (similar to stoloniferous budding) **(e.g. Plate 6, Fig. B; Plate 7, Fig. F)**.

**Scolecoid Corallum**: See Solitary Corallum.

**Septum (**pl. **Septa)**: Radially arranged longitudinal partitions of a corallite **(Figure 1)**, usually arranged in hexameral symmetry. Septa are added in **cycles**, the first cycle composed of 6 septa, the second also of 6, the third of 12, the fourth of 24, the fifth of 48, etc. resulting in corallites consisting of 6, 12, 24, 48, or 96, etc. septa per calice. Septa can bear smooth, dentate, or laciniate axial margin **(Figure 1)**.

**Solitary Corallum**: Solitary coralla exist in a variety of shapes, the shape being one of the criteria used to differentiate genera and species. Many solitary coralla are shaped as an inverted cone (**conical**), and may be attached and straight or free and usually curved. If the edges of the cone diverge at a hypothetical basal angle of 10–40°, this corallum is called **ceratoid (e.g. Plate 10, Figs H–I)**, if the angle is 40–60°, then **trochoid (e.g. Plate 8, Fig. F)**, if the angle is 60–80°, **turbinate (e.g. Plate 13, Fig. H),** and if the angle is160–180° and the corallum is low, **discoidal (e.g. Plate 18, Fig. P)**. Coralla may also be **cylindrical (e.g. Plate 22, Fig. N)**, and if the cylinder is irregular in shape, **scolecoid (e.g. Plate 12, Fig. D)**. Others are wedge-shaped (**cuneiform**) or **bowl-shaped (e.g. Plate 17, Figs F, H, J, M)**. Still others have a flat base with a convex upper surface (**cupolate**) **(e.g. Plate 19, Figs A–F)** and others are simply onion-shaped or irregular (**globular**) **(e.g. Plate 18, Fig. B)**.

**Spongy Columella**: See Columella.

**Stereome**: A general term for thick calcareous deposits, generally thickening various parts of the corallum.

**Stoloniferous Budding**: A type of extratentacular budding in which polyps are asexually generated from a thin, elongate, encrusting coenenchymal ribbon, the connecting ribbon often obscured by encrusting organisms **(e.g. Plate 5, Fig. B; Plate 6, Fig. D)**.

**Styliform Columella**: See Columella.

**Synapticular Plate**: Ribbons of calcium carbonate linking adjacent fungiacyathid septa, first appearing as vertical rods midway between septa, later bifurcate, the two ends fusing to adjacent septal faces (T- or Y-shaped) (Cairns 1989a) **(e.g. Plate 25, Fig. F)**.

**Trabecular Columella**: See Columella.

**Tabular Endothecal Dissepiment**: See Dissepiment.

**Tectura**: See Epitheca.

**Theca (Figure 1)**: The skeletal sides, or walls, of solitary coralla or corallites of colonial corals, that enclose the polyps. If the theca is solid, it is termed **imperforate (e.g. Plate 9, Figs D, L)**; if the theca is porous, **perforate (e.g. Plate 22, Figs I–J)**.

**Thecal spots/pores**: Some genera have longitudinal series of small pores aligned with the interseptal spaces of various cycles, termed thecal pores. In other genera, these analogous structures do not penetrate the theca but are visible only as slightly differently colored spots of a constructional consistently different from the remaining theca, these termed spots **(e.g. Plate 11, Figs F–G; Plate 22, Fig. N)**.

**Thecal spur**: See Edge Spine.

**Trabecular Columella**: See Columella.

**Transverse Division**: One of the three main methods of asexual reproduction among the Scleractinia (Cairns 1988b). In this method an attached sexually produced solitary corallum (called the **anthocaulus - e.g. Plate 25, Figs H, J**) transversely divides it corallum usually along a crescent-shaped line of thecal weakness, producing an asexually generated distal corallum (the **anthocyathus - e.g. Plate 25, Figs G, I**), which falls to the substrate as an unattached (free) corallum, the base of which retains a scar of previous attachment **(e.g. Plate 25, Figs I–J)**.

**Trochoid Corallum**: See Solitary Corallum.

**Turbinate Corallum:** See Solitary Corallum.

**Zooxanthellate**: Species that have symbiotic relationships with unicellular photosynthetic dinoflagellates (*Symbiodinium* spp.).
